# Carbon Monoxide Nanomodulator Reverses Ischemia‐Reperfusion Injury in Stroke: A Novel Dual‐Channel Therapy Mode of Co‐driving Neuroprotection and Neurogenesis

**DOI:** 10.1002/advs.202512333

**Published:** 2025-10-17

**Authors:** Xuegang Niu, Bin Gao, Hongyi Huang, Zesheng Li, Yibin Zhang, Quanlei Liu, Chao Zhang, Yang Dai, Jinkun Xu, Mingshan Liu, Yuanyuan Zhang, Yihe Wang, Penghu Wei, Yuanxiang Lin, Yongzhi Shan, Yumin Luo, Dezhi Kang, Guoguang Zhao

**Affiliations:** ^1^ Department of Neurosurgery Xuanwu Hospital Capital Medical University Beijing 100053 China; ^2^ Department of Neurosurgery Neurosurgery Research Institute The First Affiliated Hospital Fujian Medical University Fuzhou 350005 China; ^3^ Fujian Provincial Institutes of Brain Disorders and Brain Sciences The First Affiliated Hospital Fujian Medical University, Fuzhou Fujian 350005 China; ^4^ Beijing Geriatric Medical Research Center Beijing 100053 China; ^5^ National Medical Center for Neurological Diseases Beijing 100053 China

**Keywords:** dual‐channel therapy, ischemia‐reperfusion injury, ischemic stroke, minimizing brain injury, restoring neurofunctions

## Abstract

Recanalization intervention has improved patient outcomes in ischemic stroke, but severe ischemia‐reperfusion injury remains a major challenge, necessitating effective pharmacotherapy to reverse neuronal damage and recover neurofunctions. Traditional neuroprotection strategies aim to inhibit neuronal death, and are still insufficient to recover long‐term neurological dysfunctions. In this work, it is found that carbon monoxide (CO) as a neuromodulator exerts a new role in promoting neurogenesis via the crosstalk between brain endothelial cells and neural stem cells, which is beyond its recognized roles in anti‐inflammation and anti‐oxidation. This reveals a new possibility to address the above challenge. Furthermore, this work develops a biomimetic and reactive oxygen species‐activated CO nanogenerator to effectively penetrate blood‐brain barrier, arrive in stroke‐affected regions, and release CO in a controlled manner for an innovative dual‐channel therapy strategy via co‐driving neuroprotection and neurogenesis. This strategy further demonstrates its therapeutic effects on reversing brain injury and recovering neurofunctions in a mouse ischemic stroke model. This work reveals an important new role of CO, and further offers an advanced pharmacotherapy for long‐term neurological dysfunctions in ischemic stroke.

## Graphical abstract

This work develops a biomimetic and reactive oxygen species‐activated carbon monoxide (CO) nanogenerator to penetrate blood‐brain barrier, arrive in stroke‐affected regions, and release CO in a controlled manner for an innovative dual‐channel therapy strategy via co‐driving neuroprotection and neurogenesis.

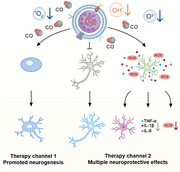

## 1 Introduction

Ischemic stroke, resulting from cerebral artery occlusion and subsequent blood and oxygen depletion, is the most prevalent stroke subtype, accounting for >10 million new cases annually worldwide.^[^ ^1^, ^2^, ^3^ ^]^ This has contributed to high mortality rates and long‐term disability, posing a significant public health challenge. Current clinical therapies, i.e., intravenous thrombolysis and intra‐arterial thrombectomy, aim to restore blood supply, but inevitably trigger the secondary damage to vulnerable neurons primarily mediated by excessive oxidative stress and neuroinflammation, referred to as ischemia‐reperfusion injury.^[^ ^4^, ^5^, ^6^ ^]^ Existing pharmaceutical interventions are hardly to modify the progression of ischemia‐reperfusion injury in clinical settings, and only acquire the limited efficacy. Therefore, advanced pharmacotherapy should be urgently explored to not only minimize brain injury, but also improve long‐term neurological dysfunctions, which remain the biggest obstacle in this research field.^[^ ^7^, ^8^ ^]^

Recently, neuroprotection strategies are highlighted to reduce neuronal death and provide a favorable microenvironment, i.e., applying cell‐death inhibitors to promote neuronal survival and reduce neuronal apoptosis, and antioxidant/anti‐inflammatory agents and their delivery nanosystems to alleviate oxidative stress/neuroinflammation.^[^ ^9^, ^10^, ^11^, ^12^, ^13^, ^14^ ^]^ While these neuroprotective strategies have shown promise in preclinical studies, they are still insufficient to improve the long‐term neurological dysfunctions following ischemic stroke due to the limited self‐repair capacity of central nervous system.^[^ ^15^ ^]^

Neurogenesis is an endogenous process of forming new neurons from neural stem cells (NSC) throughout the life. The newly formed neurons preferentially migrate to the damaged brain region and integrate into neural networks, which play an essential role in neurofunctional recovery. However, endogenous neurogenesis typically occurs at a low rate, and additional strategies focus on enhancing neurogenesis to improve long‐term neurofunctions.^[^ ^15^ ^]^ Unfortunately, it is estimated that ≈50%–80% of newly generated neurons from neurogenesis events face the fate of death following ischemic stroke, highlighting the critical challenge of ensuring the survival of these neurons.^[^ ^16^ ^]^

Therefore, we propose that an innovative dual‐channel therapeutic strategy based on integrating neuroprotection with the enhanced neurogenesis, which is considered as a potential approach to addressing the challenges of minimize brain injury and long‐term neurological dysfunctions. This mainly benefit from the following advantages: 1) Neuroprotection improves the survival of newly generated neurons; 2) The enhanced neurogenesis further increases neuron number in lesions; 3) Their collaboration potentially minimizes brain damage and accelerates neurofunctional recovery. Thereby, how to discover a dual‐channel therapeutic molecule that can simultaneously modulate neuroprotection and neurogenesis is crucial to open the effective pharmacotherapy of cerebral ischemia‐reperfusion injury in stroke.

Accumulating evidence suggest that CO plays multiple physiological roles as a second messenger at low levels, i.e., inhibiting the oxidative stress and inflammatory responses, as well as increasing cell survival, which has exhibited therapeutic potentials delivered by nanoparticles in multiple diseases.^[^ ^17^ ^]^ These also predict its neuroprotective effects.^[^ ^18^, ^19^, ^20^ ^]^ But whether CO has an additional role in modulating neurogenesis? This work finds a new role of CO in promoting neurogenesis through the brain endothelial cells‐NSC crosstalk, which provides a unique research perspective to develop dual‐channel pharmacotherapy. To implement this therapy, several challenges still need to be addressed: 1) The efficient delivery of CO to ischemic lesion after penetrating blood‐brain barrier (BBB); 2) The controlled release of CO at low levels to ensure therapeutic performance and avoid toxicity risks at high levels.

**Figure 1:**
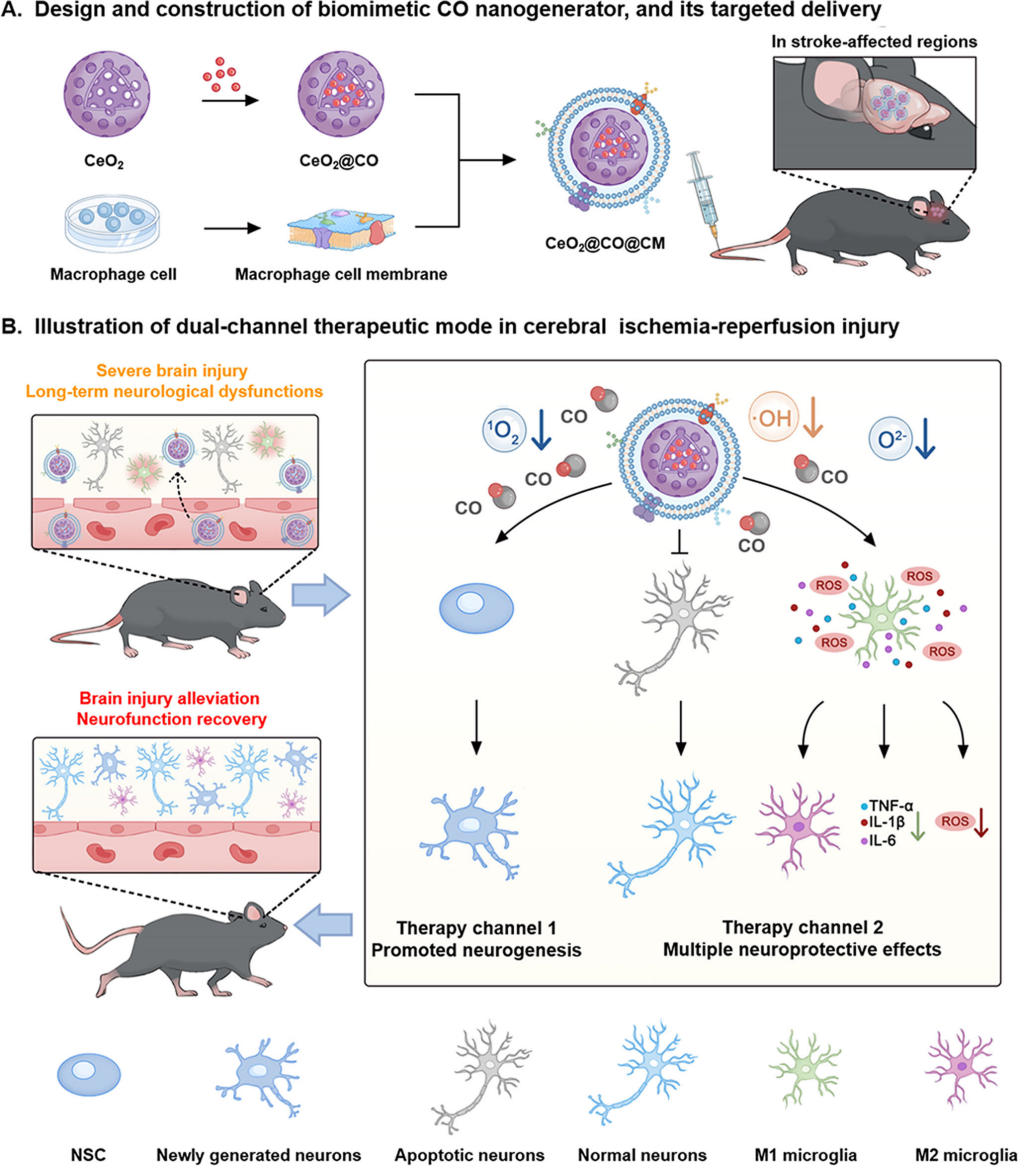
Schematic illustration of biomimetic CO nanogenerator for an innovative dual‐channel stroke therapy via integrating neuroprotection with enhanced endogenous neurogenesis. A) Design and construction of biomimetic CO nanogenerator, as well as its delivery capability of penetrating blood‐brain barrier and arriving in stroke‐affected site upon intravenous administration. B) The biomimetic CO nanogenerator reduced the microglial cells‐mediated neuroinflammation/oxidative stress and inhibited neuronal apoptosis for exerting multiple neuroprotective effects, also promoted proliferation and differentiation of NSC in SVZ for enhancing neurogenesis, in which this dual‐channel therapeutic mode reduced brain injury and restored neurofunctions following ischemia‐reperfusion injury in stroke.

Given that CO has dual‐effects of promoting neurogenesis and neuroprotection,^[^ ^18^, ^19^, ^20^ ^]^ we therefore designed a biomimetic and ROS‐activated CO nanogenerator, where hollow mesoporous cerium dioxide nanoparticles (CeO_2_) were used to load the carbonyl manganese (MnCO, a ROS‐activated CO‐releasing molecule),^[^ ^21^ ^]^ and then camouflaged with macrophage cell membrane to formulate this biomimetic and ROS‐activated CO nanogenerator (CeO_2_@CO@CM) (**Figure** **1**A). CeO_2_ was selected based on the two main reasons: 1) excellent ROS‐scavenging property to enhance neuroprotective effects; 2) hollow mesoporous structure to offer abundant drug‐loading sites. Besides, the MnCO was utilized resulting from its ROS‐susceptibility to control CO release according to severity of ischemia‐reperfusion injury. And coating of macrophage cell membrane enabled CO nanogenerator to preferentially penetrate BBB and arrive in stroke‐affected regions mediated by the inflammatory chemotaxis of macrophages.^[^ ^22^, ^23^ ^]^ Consequently, as shown in Figure 1B, in a mouse ischemic stroke model, this biomimetic CO nanogenerator could effectively penetrate BBB and distribute in brain lesion to reduce neuroinflammation/oxidative stress and inhibit neuronal apoptosis for exerting neuroprotective effects, also to promote the proliferation and differentiation of NSC in the subventricular zone (SVZ) for enhancing neurogenesis. Consequently, this dual‐channel therapeutic mode reduced brain injury and restored neurofunctions. Our work not only revealed an important new role of CO, but also offered an advanced dual‐channel pharmacotherapy to alleviate the concerns regarding high mortality rate and long‐term disability following cerebral ischemia‐reperfusion injury.

## 2 Results and Discussion

### 2.1 CO Promoted Neurogenesis via Modulating Brain Endothelial Cell‐Neural Stem Cell Crosstalk

**Figure 2:**
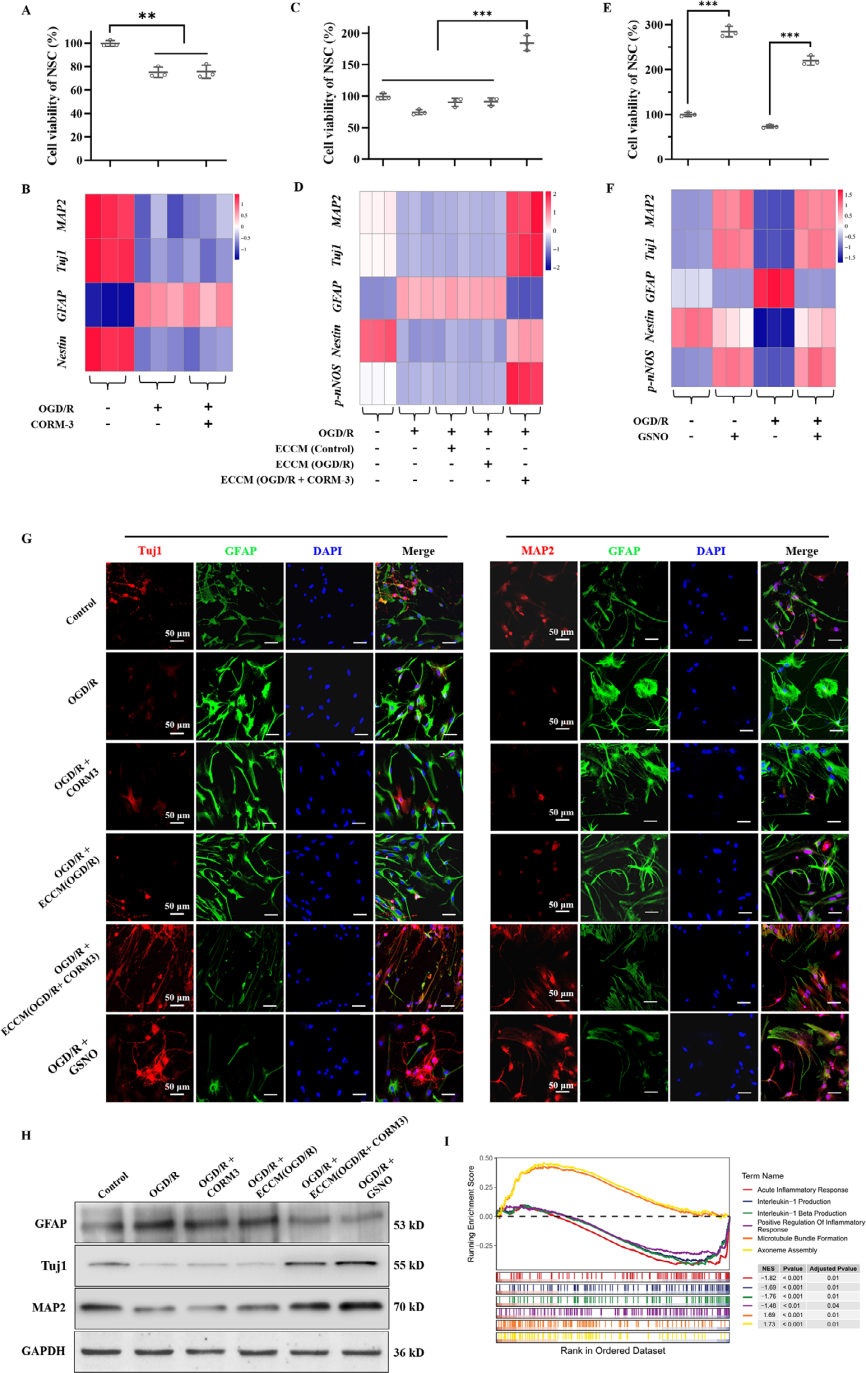
CO promoted neurogenesis via modulating BMEC‐NSC crosstalk. A,C,E) CCK‐8 assays to examine NSC counts under different conditions for assessing the proliferation of NSC. B,D,F) RT‐PCR assays to examine relative gene expressions of *MAP2*, *Tuj1*, *GFAP*, *Nestin*, and *p‐nNOS* in NSC under different conditions for assessing the differentiation of NSC. G) Cell immunostaining assays to study the expressions of Tuj1 (red)/GFAP (green)/DAPI (blue), MAP2 (red)/GFAP (green)/DAPI (blue) in NSC under different conditions for assessing the differentiation of NSC. H) Western blot assay to examine the protein expression of MAP2, Tuj1, GFAP in NSC under different conditions for assessing the differentiation of NSC. I) GSEA of Gene Ontology biological processes in bulk RNA‐sequencing. The *x*‐axis representing rank of genes ordered by differential expression (ECCM treatment group subjected to OGD/R and CORM‐3 versus control group), and the *y*‐axis denoting the running enrichment score (ES), with the dashed horizontal line indicating ES = 0. Barcode plots beneath each curve marking the positions of all member genes in the ranked list. The table on the right reports, for each gene set, the normalized enrichment score (NES), nominal *p*‐value and FDR‐adjusted *q*‐value. Data were presented as mean ± SEM. Statistical comparisons between two groups were performed using an unpaired two‐tailed Student's *t*‐test, and one‐way ANOVA test was applied for comparisons among multiple groups. **P < 0.01, ***P < 0.001.

First, this work focuses on investigating whether the CO has a biological effect on modulating neurogenesis, which is expected as a basis for developing a dual‐channel therapy. As a second messenger, endogenous gas signaling molecule CO participates in various physiological and pathological processes. In this section, we aim to examine whether CO could exert an additional modulatory effect on neurogenesis. First, we subjected primary mouse NSC to the oxygen‐glucose deprivation/reperfusion (OGD/R) model. Under this condition, cell counting kit‐8 (CCK‐8) result confirmed that NSC counts were reduced (**Figure** **2**A). And then, incubation with CO‐releasing molecule 3 (CORM‐3, a water‐soluble CO‐releasing molecule that is commonly used to study effect of CO on cellular systems^[^ ^23^ ^]^) did not rescue NSC counts or induce any significant changes in directed‐neuronal differentiation of NSC, as the expression of NSC marker *Nestin*, and neuron‐specific markers (neuronal marker class III beta‐tubulin (*Tuj1*), an early marker of neuronal differentiation; neuronal marker microtubule associated protein‐2 (*MAP2*), a marker of mature neuronal differentiation) remained unchanged in RT‐PCR assays. (Figure 2A,B). The concept of neurovascular unit emphasized the importance of cell‐cell signaling between neural, glial and vascular compartments.^[^ ^24^, ^25^ ^]^ Brain microvascular endothelial cells (BMEC) played an essential role in supporting the neurogenesis and angiogenesis.^[^ ^26^ ^]^ Hence, we further investigated whether CO indirectly modulated neurogenesis via BMEC‐NSC crosstalk. In Figure 2B, it was observed that OGD/R made NSC toward an astrocyte‐like differentiation fate, as evidenced by the increased glial fibrillary acidic protein (*GFAP*) expression. In addition, the addition of BMEC‐conditioned medium (ECCM) with or without OGD/R didn't modify this outcome of astrocyte‐like differentiation (Figure 2C,D). Interestingly, the ECCM group (subjected to OGD/R and CORM‐3 pretreatment) could increase the NSC counts and enhance the directed differentiation of NSC into mature neurons, reflected by the increased *Tuj1* and *MAP2* expressions (Figure 2C,D). In the meanwhile, the glial differentiation of NSC was inhibited as the *GFAP* expression reduced. The findings illustrated that CO could indirectly promote neurogenesis via modulating BMEC‐NSC crosstalk. Furthermore, it was detected that BMEC could increase the gene expression of endothelial nitric oxide synthase (eNOS) and the production of nitric oxide (NO) upon CORM‐3 treatment (Figures S1 and S2, Supporting Information). This indicated that CORM‐3 could promote the eNOS‐NO signaling in BMEC to secret NO. Further, it was discovered that the NSC was accompanied by an increase in the expression of phosphorylated neuronal nitric oxide synthase (*p‐nNOS*), which was beneficial to enhance the neuronal development.^[^ ^27^ ^]^ To elucidate this mechanism, we directly added the NO donor (Nitrosoglutathione, GSNO) to NSC at either normal condition or OGD/R condition, finding that it could increase expression of *p‐nNOS*, facilitate the directed‐neuronal differentiation of NSC, and increase the NSC counts (Figure 2E,F). Collectively, these results preliminarily illustrated that CO could promote NO signaling transduction between BMEC and NSC for the enhanced neurogenesis.

To further confirm this result, several experiments were also conducted. First, cell immunostaining and western blot were performed to study differentiation behavior of NSC. As shown in Figure 2G,H, the ECCM group (subjected to OGD/R and CORM‐3 pretreatment) could indeed enhance the expressions of neuron‐specific markers Tuj1 and MAP2, and inhibit GFAP expression, indicating directed‐neuronal differentiation of NSC. Besides, direct NSC treatment with NO donor GSNO also had similar effects, further verifying the fact that CO enhanced neurogenesis via promoting NO signaling transduction between BMEC and NSC. In addition, we performed RNA‐sequencing experiment on NSC at the OGD/R condition with and without ECCM treatment group (subjected to OGD/R and CORM‐3 pretreatment). The GSEA of Gene Ontology biological processes revealed that this ECCM treatment not only upregulated several pathways associated with the directed‐neuronal differentiation and maturing (i.e., microtubule bundle formation and axoneme assembly), but also downregulated several pathways relevant to neuroinflammatory responses (i.e., acute inflammatory responses, interleukin‐1 production, interleukin‐1 beta production, positive regulation of inflammatory response, pointing to the dual‐effects of CO on enhancing neurogenesis and inhibiting neuroinflammation (Figure 2I).

### 2.2 Preparation and Characterizations of Biomimetic CO Nanogenerator

**Figure 3:**
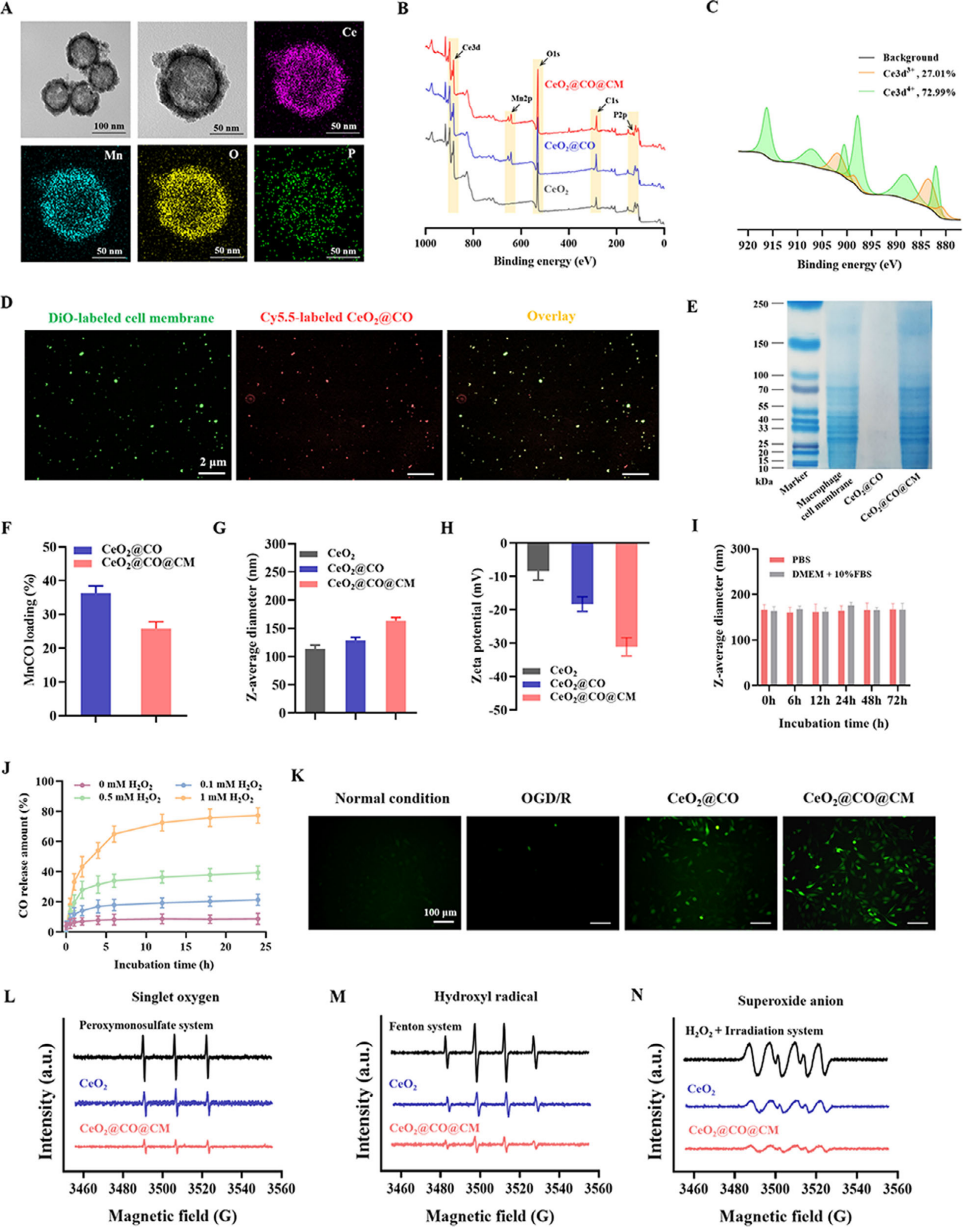
Preparation and characterizations of biomimetic CO nanogenerator. A) TEM image and elemental mapping to detect the microscopic morphology and elemental composition of CeO_2_@CO@CM. B) XPS spectra of CeO_2_, CeO_2_@CO, and CeO_2_@CO@CM. C) High‐resolution XPS analysis of Ce3d showing the binding energy levels of Ce3d^3+^ and Ce3d^4+^. D) CLSM visualizing the overlay of CeO_2_@CO and macrophage cell membrane. E) Sodium dodecyl sulfate polyacrylamide gel electrophoresis (SDS‐PAGE) examining the protein bands of macrophage cell membrane coating. F) MnCO loading content of CeO_2_@CO, CeO_2_@CO@CM. G,H) Z‐average diameter and zeta potential of CeO_2_@CO@CM in PBS (10 × 10^−3^ m, pH = 7.4) tested by Zetasizer Nano ZS. I) Colloidal stability of CeO_2_@CO@CM in different biological media. J) CO release amount of CeO_2_@CO@CM under different concentrations of H_2_O_2_. K) Intracellular CO level visualized by CO fluorescence probe upon various treatments. Scale bars: 100 µm. L–N) The ROS‐scavenging property of CeO_2_@CO@CM analyzed by EPR, including singlet oxygen, hydroxyl radical, superoxide anion. Data were presented as mean ± SEM.

Based on the above experimental results, it was confirmed that CO could promote neurogenesis. In addition, literatures revealed the potential of CO in neuroprotection.^[^ ^18^, ^19^, ^20^ ^]^ Therefore, it was rationally speculated that CO possessed dual‐effects of promoting neurogenesis and neuroprotection, which was crucial for developing the dual‐channel therapeutic strategy in ischemic stroke. To implement this therapy, we further constructed a biomimetic and ROS‐activated CO nanogenerator CeO_2_@CO@CM in this work. Specifically, the CeO_2_ nanoparticle was used to encapsulate MnCO (ROS‐activated CO‐releasing molecule) to form CO nanogenerator (CeO_2_@CO), then it was physically coated by macrophage cell membrane to formulate this biomimetic CO nanogenerator (CeO_2_@CO@CM). To characterize this preparation, the high‐resolution transmission electron microscopy (TEM) was firstly performed. Morphology results showed that the CeO_2_@CO@CM was spherical nanoparticles with the hollow structures, and exhibited the relatively thicker surface with the cell membrane‐coating characteristics (**Figures** **3**A and S3, Supporting Information). Furthermore, the elemental mapping results detected the typical Ce element from CeO_2_, the Mn element from MnCO molecule, and the P element from cell membrane (Figure 3A), implying the successful macrophage cell membrane coating. Besides, X‐ray photoelectron spectroscopy (XPS) tests also found the Mn and P elements after MnCO loading and cell membrane coating (Figure 3B). These results supported that this two‐step preparation process was successfully conducted to prepare biomimetic CO nanogenerator. Moreover, the valent state of ceria was analyzed by high‐resolution XPS spectra, showing the presence of Ce^3+^ (peaks at ≈880.84, 883.42, 898.44, 901.81 eV) and Ce^4+^ (peaks at ≈882.00, 888.13, 897.81, 900.51, 907.03, 916.20 eV) (Figure 3C). The quantified result revealed that CeO_2_@CO@CM contained ≈27.01% Ce^3+^ and ≈72.99% Ce^4+^, confirming the mixed‐valent state of ceria for providing a structural basis of circulating ROS‐scavenging property.^[^ ^28^, ^29^ ^]^ In addition, we applied DiO dyes to label macrophage cell membrane and Cy5.5 dyes to label CeO_2_@CO. After conducting cell membrane coating process, the confocal laser scanning microscopy (CLSM) was performed to observe a high co‐localization between cell membrane and CeO_2_@CO, which provided direct evidences for CeO_2_@CO@CM preparation (Figure 3D). And, CeO_2_@CO@CM retained protein bands from macrophage cell membrane by sodium dodecyl sulfate polyacrylamide gel electrophoresis (SDS‐PAGE) (Figure 3E). And CeO_2_@CO@CM had ≈25.3% loading content of MnCO by Inductively Coupled Plasma Optical Emission Spectrometer (ICP‐OES) (Figure 3F). Subsequently, colloid properties were tested in aqueous medium by Zetasizer Nano ZS, showing that the initial CeO_2_ nanoparticle had ≈113.7 nm of Z‐average diameter and ≈−8.4 mV of zeta potential, while CeO_2_@CO exhibited ≈128.7 nm of Z‐average diameter and ≈−18.3 mV of zeta potential (Figure 3G,H). By comparison, this increased particle size also implied the fact of MnCO loading. Besides, CeO_2_@CO@CM showed ≈163.3 nm of Z‐average diameter and ≈−31.1 mV of zeta potential, illustrating that negatively charged cell membrane was coated onto CeO_2_@CO surface with ≈35 nm of coating thickness. In addition, it was noticed that particle sizes of CeO_2_@CO@MP maintained good colloidal stability in PBS (10 × 10^−3^ m, pH = 7.2–7.4) or DMEM + 10% fetal bovine serum (FBS) within 72 h (Figure 3I).

We continued to explore the physicochemical properties of CeO_2_@CO@CM. First, CO fluorescent probe was synthesized according to our previous work (Figure S4, Supporting Information),^[^ ^30^ ^]^ which was used to study the CO‐releasing profile, showing that CO was released in a ROS‐dependent manner, and cumulative release rate within 24 h reached ≈77.3% at 1 × 10^−3^ m H_2_O_2_, and only ≈8.7% without H_2_O_2_ incubation (Figure 3J). This reflected that this biomimetic CO nanogenerator could maintain stable in normal condition and activate CO‐releasing mode in ischemic lesion due to ROS upregulation. Furthermore, we also used CO fluorescent probe to visualize the CO‐releasing in the cell model of OGD/R, showing that CeO_2_@CO@CM could release the gas signaling molecule CO in hippocampal neuronal cells (HT22 cells) pretreated with OGD/R (Figure 3K). Afterward, we also investigated its ROS‐scavenging property, which was mainly attributed to two components. One was CeO_2_ nanoparticle with persistent ROS‐scavenging behavior due to the reversible conversion between Ce^3+^ and Ce^4+^. Another one was MnCO molecule that could deplete ROS components to generate CO. Electron paramagnetic resonance (EPR) was performed to detect the scavenging capability to multiple ROS components, including singlet oxygen, hydroxyl radical, and superoxide anion. It was detected that CeO_2_ nanoparticle could effectively inhibit generations of singlet oxygen, hydroxyl radical, and superoxide anion. And the additions of MnCO and cell membrane further enhanced ROS‐scavenging effect (Figure 3L–N). Moreover, the 2,2‐diphenyl‐1‐picrylhydrazyl (DPPH) and the 2,2′ azobis (3‐ethylbenzothiazoline‐6‐sulfonic acid) (ABTS) assays were performed to assess total antioxidant capacity of CeO_2_@CO@CM. Results indicated that CeO_2_ nanoparticle could inhibit the formations of DPPH and ABTS free radicals in a dose‐dependent manner, and then this inhibitory effect was augmented by CeO_2_@CO@CM with >60% of scavenging efficiency at ≈50 µg mL^−1^ for DPPH assay, and at ≈20 µg mL^−1^ for ABTS assay (Figures S5 and S6, Supporting Information). These results proved that our designed biomimetic CO nanogenerator had an excellent ROS‐scavenging property.

### 2.3 In Vitro Neuroprotective Effects and Safety

**Figure 4:**
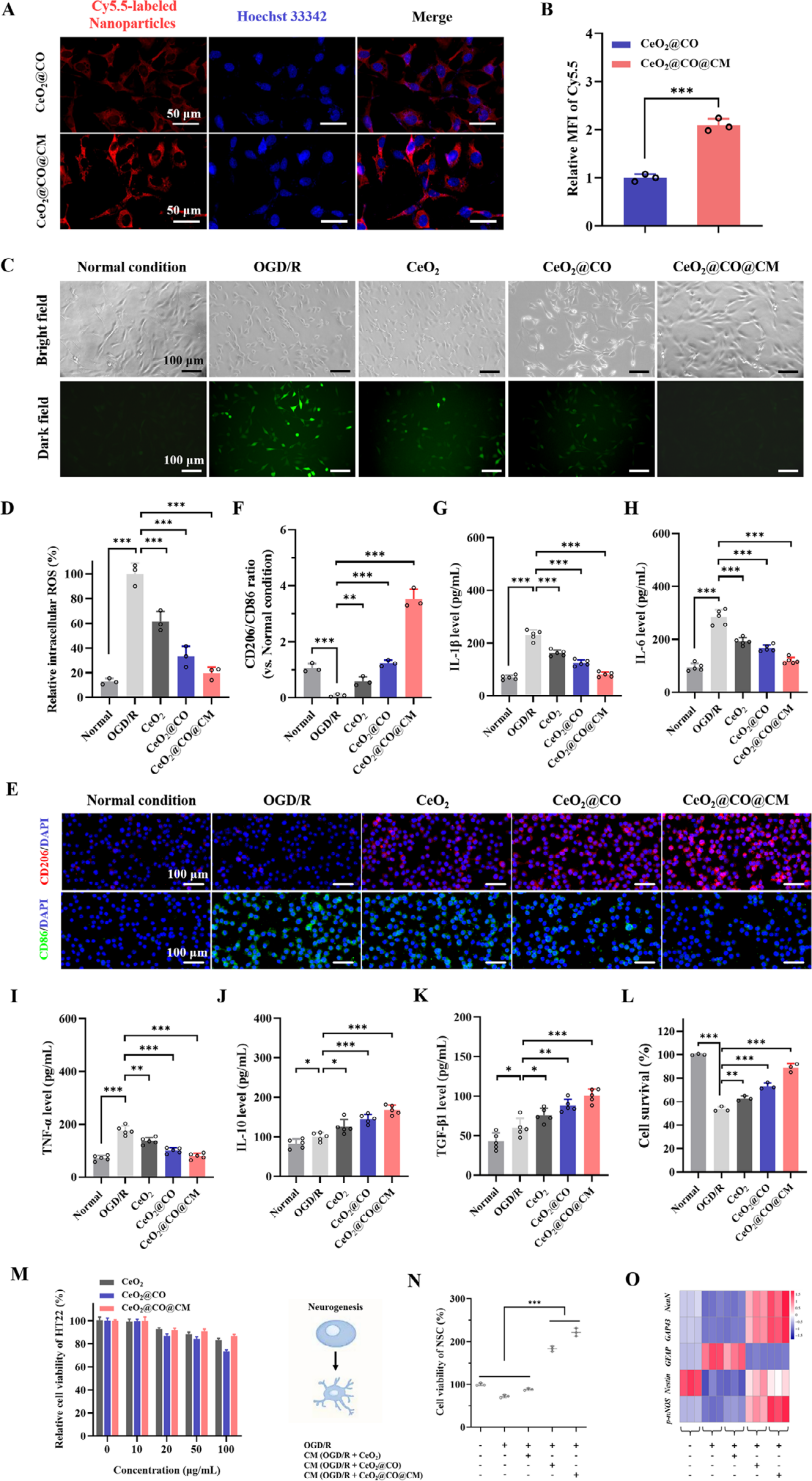
In vitro evaluations on neuroprotective and neurogenesis‐enhancing effects. A,B) The CLSM visualizing the cellular uptake of CeO_2_@CO and CeO_2_@CO@CM in Bend.3 cells, and quantitative analysis of mean fluorescence intensity (MFI) reflecting the level of cellular uptake. Scale bars: 50 µm. C,D) Intracellular ROS visualized by DCFH‐DA probe upon various treatments, and quantitative analysis of MFI reflecting intracellular ROS level. Scale bars: 100 µm. E,F) Immunofluorescence staining of CD206 and CD86 reflecting the polarization situation of BV2 cells upon various treatments, and quantitative analysis of CD206/CD86 ratio assessing M2 polarization level. Scale bars: 100 µm. G–I) The levels of the M1‐type cytokines in BV2 cells upon various treatments examined by ELISA assay, including IL‐1β, IL‐6, and TNF‐α. J,K) The levels of the M2‐type cytokines in BV2 cells upon various treatments examined by ELISA assay, including IL‐10 and TGF‐β1. L) Neuronal survival upon various treatments examined by CCK‐8 assay. M) Cytotoxicity of CeO_2_@CO@CM to HT22. N) CCK‐8 assay to examine NSC counts upon various treatments for assessing the proliferation of NSC. O) RT‐PCR assay to examine relative gene expressions of *MAP2*, *Tuj1*, *GFAP, Nestin*, and *p‐nNOS* upon various treatments for assessing the differentiation of NSC. Data were presented as mean ± SEM. Statistical comparisons between two groups were performed using an unpaired two‐tailed Student's *t*‐test, and one‐way ANOVA test was applied for comparisons among multiple groups. **P < 0.01, ***P < 0.001.

After demonstrating physicochemical properties of CeO_2_@CO@CM, we further studied its performance in neuroprotective effects and safety in vitro. This biomimetic CO nanogenerator could not only scavenge ROS but also release CO in a controlled manner, which was speculated as a potential neuroprotective agent. Herein, we investigated its neuroprotective effects in vitro. First, Cy5.5‐labeled CO nanogenerators were incubated with the mouse BMEC (Bend.3 cells) pretreated with OGD/R. Then, CLSM imaging observation revealed that CeO_2_@CO@CM and CeO_2_@CO could be internalized into Bend.3 cells, and the cellular uptake of CeO_2_@CO@CM was ≈2.1‐fold in comparison with that of CeO_2_@MnCO, suggesting that the coating of macrophage cell membrane endowed CO nanogenerator with the enhanced affinity with Bend.3 cells (**Figure** **4**A,B). Subsequently, we studied the effect of CeO_2_@CO@CM on intracellular oxidative stress by using the 2,7‐dichlorodihydrofluorescein diacetate (DCFH‐DA) probe to assess intracellular ROS level.^[^ ^31^ ^]^ Green fluorescence intensity of DCFH‐DA positively correlated with the ROS level. As exhibited in Figure 4C,D, it was found that OGD/R treatment increased ROS production inside HT22 cells, whereas the CeO_2_ slightly decreased intracellular ROS level due to intrinsic ROS‐scavenging capability. However, benefiting from the dual‐mechanism of ROS‐scavenging, the CeO_2_@CO had relatively stronger capability of reducing intracellular ROS level. And this effect was further improved in CeO_2_@CO@CM group, attributing to higher internalization rate from the macrophage cell membrane. As expected, our designed biomimetic CO nanogenerator exhibited a superior ability to downregulate intracellular ROS level for alleviating oxidative stress after OGD/R treatment.

In addition, microglia‐mediated neuroinflammation was also an essential factor of poor prognosis after cerebral ischemia‐reperfusion injury.^[^ ^32^ ^]^ Therefore, we further studied effects of CeO_2_@CO@CM on microglia polarization and neuroinflammation in vitro. By immunofluorescence staining assay, we confirmed that OGD/R enabled microglia polarization toward M1 subtype because M1 marker CD86 upregulated (Figure 4E,F). This also significantly increased pro‐inflammatory cytokines levels (IL‐1β, IL‐6, and TNF‐α) tested by ELISA assays (Figure 4G–I), and thereby producing an inflammatory microenvironment. After incubation with CeO_2_@CO@CM, the related factors were remarkably modulated, including the elevated level of M2 marker CD206, the reduced secretions of M1‐type cytokines levels (IL‐1β, IL‐6, and TNF‐α), and the increased secretions of M2‐type cytokines levels (IL‐10 and TGF‐β1), whose effects were more significant than CeO_2_@CO and CeO_2_ groups (Figure 4E–K). These indicated that CeO_2_@CO@CM induced microglia polarization toward M2 subtype and alleviated neuroinflammation in vitro. After cerebral ischemia‐reperfusion injury, BBB was disrupted, which enabled peripheral immune cells to infiltrate into brain and aggravate neuroinflammation.^[^ ^33^ ^]^ During this pathological process, the highly expressed MMP‐9 played an important role in degrading extracellular matrix components and disrupting BBB.^[^ ^34^ ^]^ Consequently, we evaluated the effect of CeO_2_@CO@CM on BBB disruption. By immunofluorescence staining assay, we confirmed that CeO_2_@CO@CM had the strongest inhibition on MMP‐9 expression in comparison with other groups due to the dual‐effects of CO‐releasing and ROS‐scavenging (Figures S7 and S8, Supporting Information). These results indicated that this biomimetic CO nanogenerator effectively inhibited MMP‐9 expression and exhibited great potential to protect BBB integrity.

Finally, we explored the effect of CeO_2_@CO@CM on the survival of HT22 cells by a standard CCK‐8 assay. Results showed that OGD/R made cell survival rate decrease from 100% to ≈54%. And incubation with CeO_2_@CO@CM significantly saved cell survival with a higher survival rate of 89%, in contrast with other groups, including CeO_2_ and CeO_2_@CO (Figure 4L). This indicated that the CeO_2_@CO@CM inherited the capabilities of releasing CO and scavenging ROS for superior neuroprotective effects in vitro. Moreover, the cytotoxicity was evaluated, in which different concentrations of CeO_2_@CO@CM, CeO_2_@CO, CeO_2_ were incubated with relevant cells, including mouse microglial cells (BV2), HT22 cells, and Bend.3 cells. After 24 h, these cells still achieved >85% viability when these drugs were within a high concentration of 50 µg mL^−1^ (Figure 4M; Figures S9 and S10, Supporting Information). This result implied that our designed biomimetic CO nanogenerator did not have obvious toxicity to microglial cells, neurons, and brain endothelial cells.

### 2.4 In Vitro Promotion Effect on Neurogenesis

Next, we studied the performance of CeO_2_@CO@CM in promoting neurogenesis in vitro. In this section, the BMEC‐to‐NSC medium transfer assay was performed again to assess in vitro promotion effect of CeO_2_@CO@CM on endogenous neurogenesis. Above results had confirmed that OGD/R condition made NSC differentiate toward an astrocyte‐like fate rather than mature neurons. Upon addition of various drugs, we found that CeO_2_ nanoparticles did not have a remarkable influence on NSC counts and astrocyte‐like differentiation fate as exhibited by the increased *GFAP* expression (Figure 4N,O). However, CO nanogenerators, CeO_2_@CO@CM and CeO_2_@CO, greatly promoted NSC differentiation into neurons via activating the p‐nNOS signaling pathway, elevating *Tuj1* and *MAP2* expressions and reducing GFAP expression, among which CeO_2_@CO@CM exhibited the strongest promotion effect. This indicated that CeO_2_@CO@CM could effectively reverse NSC differentiation fate from astrocytes to neurons, which could predict its pharmacological activity in promoting neurofunctional recovery following cerebral ischemia‐reperfusion injury. These results verified that CeO_2_@CO@CM had a potent promotion effect on neurogenesis in vitro via the NO signaling transduction between BMEC and NSC.

### 2.5 BBB Penetration and Targeted Delivery to Ischemic Regions

The existence of BBB largely hinders intracerebral drug delivery.^[^ ^35^ ^]^ And therefore, effective BBB penetration and targeted drug delivery to ischemic lesions have become the prerequisites for therapeutic performance. To evaluate these effects, we firstly established an in vitro BBB model to assess the BBB‐penetrating capability of CeO_2_@CO@CM, where the Bend.3 cells monolayer was added to upper chamber to simulate BBB barrier with >250 Ω cm^2^ of transendothelial electrical resistance (TEER) (Figure S11, Supporting Information), and HT22 cells were added to bottom chamber to simulate intracerebral neurons. Various Cy5.5‐labeled drugs were introduced into upper chamber, and then CLSM was applied to observe the red fluorescence signals of CeO_2_@CO@CM and CeO_2_@CO in HT22 cells after 12 h. As shown in Figure S12 (Supporting Information), it was found that mean fluorescence intensity (MFI) of CeO_2_@CO@CM was ≈1.9‐fold higher than that of CeO_2_@CO, illustrating that coating of macrophage cell membrane could facilitate trans‐endothelial transportation of CO nanogenerator.

**Figure 5:**
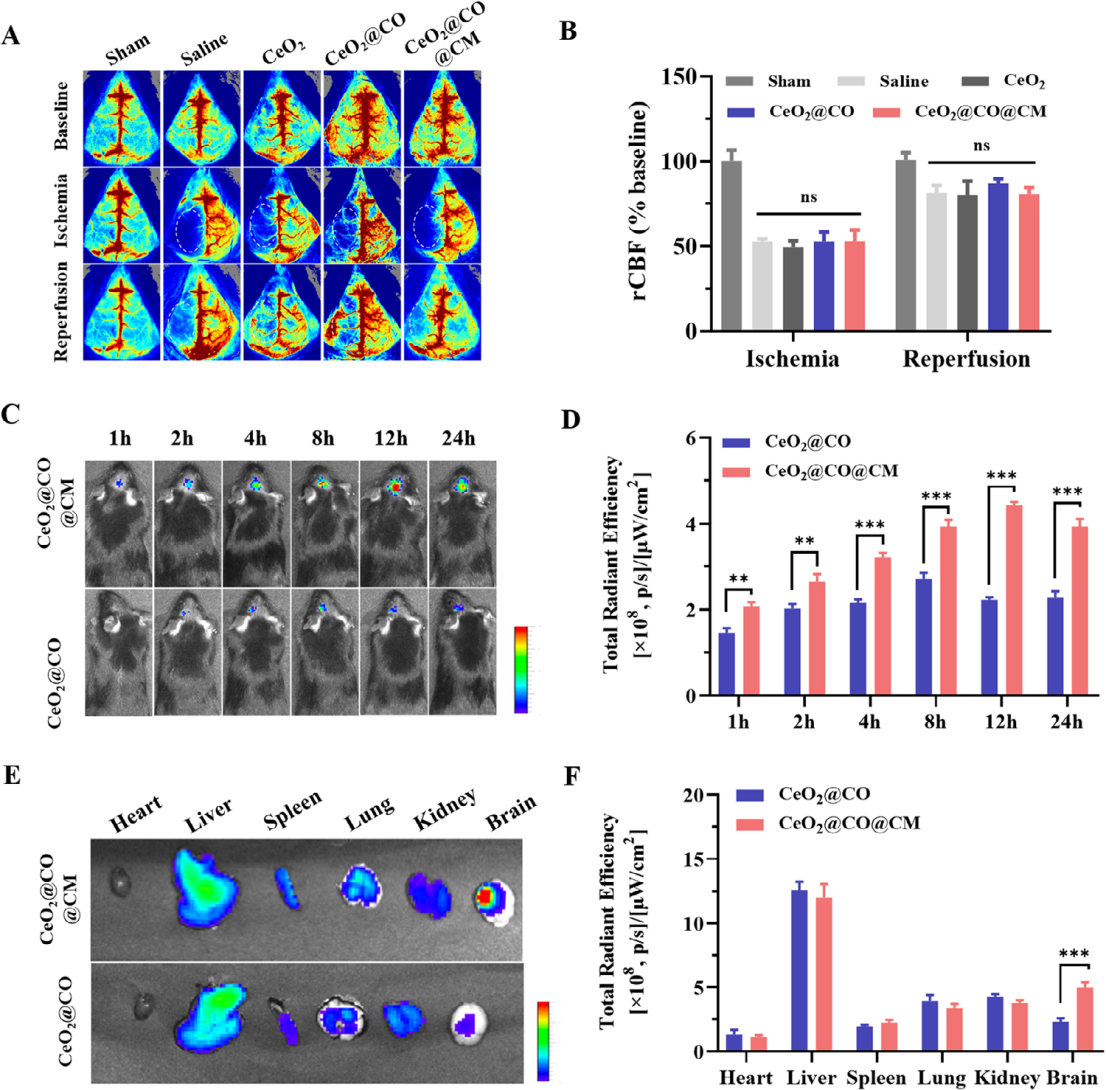
Targeted drug delivery to ischemic regions. A,B) The laser speckle contrast imaging before MCAO surgery, during surgery and after reperfusion, as well as quantitative rCBF to reflect changes of ischemia and reperfusion processes. C,D) In vivo fluorescence images of MCAO mice administrated with Cy5.5 labeled CeO_2_@CO and CeO_2_@CO@CM at different time points, and quantitative analysis of total radiant efficiency in brain. E,F) Ex vivo fluorescence images of major organs (heart, liver, spleen, lung, kidney, and brain) from Cy5.5 labeled CeO_2_@CO and CeO_2_@CO@CM at 24 h, and quantitative analysis of total radiant efficiency. Data were presented as mean ± SEM. Statistical comparisons between two groups were performed using an unpaired two‐tailed Student's *t*‐test, and one‐way ANOVA test was applied for comparisons among multiple groups. ns: not significant; **P < 0.01, ***P < 0.001.

Furthermore, we assessed the targeted drug delivery to ischemic regions using a transient middle cerebral artery occlusion (MCAO) model in mice, where laser speckle contrast imaging was conducted to monitor the regional cerebral blood flow (rCBF) (**Figure** **5**A,B). Ischemia stage could obviously reduce the blood supply to ≈50% of baseline, and then reperfusion stage further increased blood supply to ≈80% of baseline. There were no significant differences in rCBF for various treatments in ischemia and reperfusion stages, ensuring the consistency of ischemia and blood flow recovery in MCAO surgery. After MCAO, Cy5.5‐labeled CeO_2_@CO@CM and CeO_2_@CO were intravenously injected for the analysis of IVIS fluorescence imaging system. It was observed that fluorescence signals of these two groups occurred in the brain within 2 h and maintained a detectable state for 24 h (Figure 5C). However, quantitative fluorescence intensity in CeO_2_@CO@CM group was significantly higher than that in CeO_2_@CO group, suggesting that this biomimetic CO nanogenerator had a stronger ability to cross BBB and enter brain parenchyma (Figure 5D). Subsequently, the primary organs were also isolated at 24 h for *ex vivo* fluorescence imaging, which further verified this point. In Figure 5E,F, it was additionally noticed that the CeO_2_@CO@CM mainly distributed in ischemic hemisphere (left brain) rather than normal hemisphere (right brain), and thereby achieving the targeted delivery to ischemic regions and increasing bioavailability. Also, a portion of CeO_2_@CO@CM distributed in liver and spleen, indicating that it might be either cleared by the liver or metabolized by the urinary system following efficacy exertion.

### 2.6 In Vivo Neuroprotective Effects

**Figure 6:**
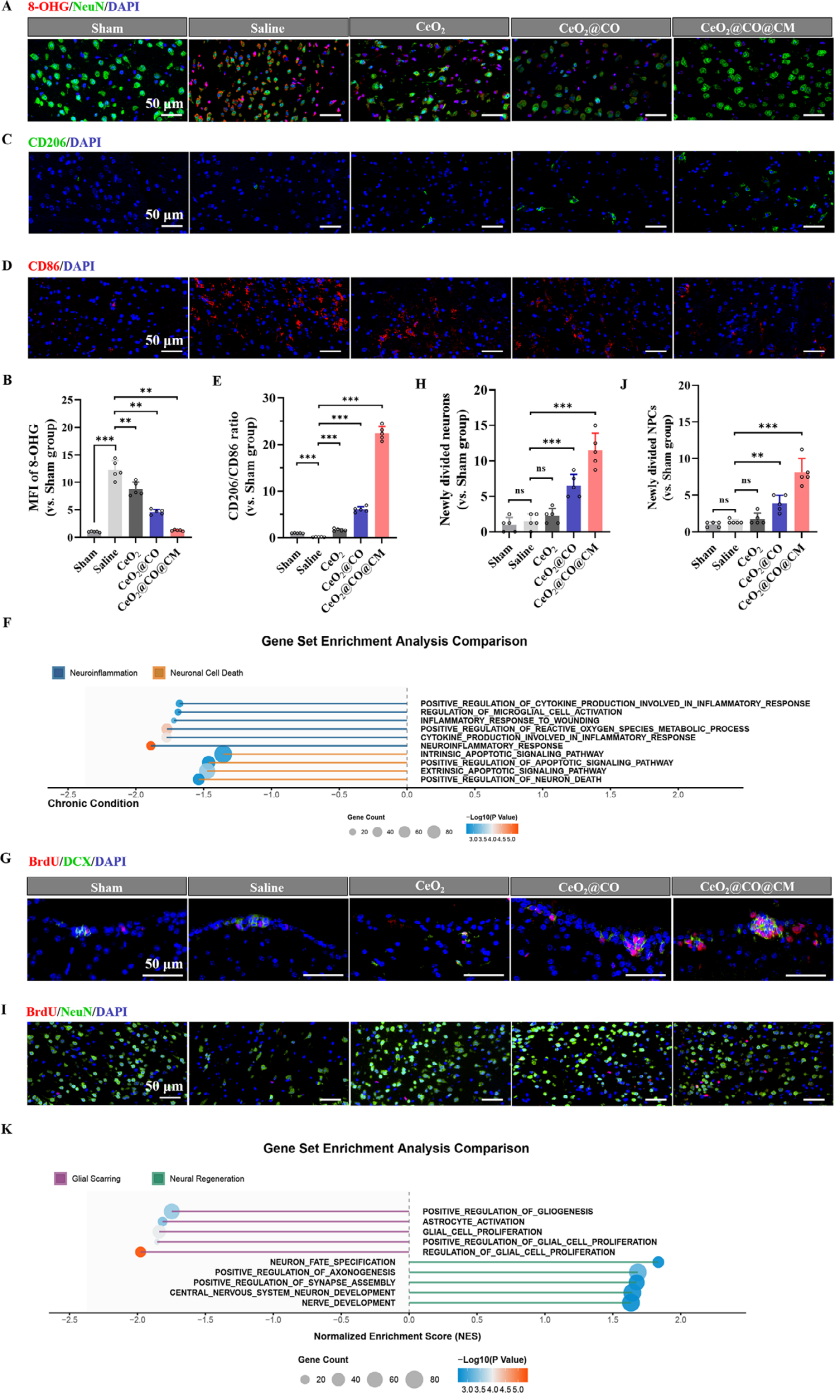
In vivo neuroprotective protections and neurogenesis‐enhancing effects. A,B) Representative immunofluorescence staining of 8‐OHG (red)/NeuN (green)/DAPI (blue) showing oxidative damage in the penumbra upon various treatments at 24 h post‐MCAO, and quantitative analysis of MFI of 8‐OHG. Scale bars: 50 µm. C–E) Representative immunofluorescence staining of CD206 (green)/DAPI (blue) and CD86 (red)/DAPI (blue) reflecting microglia polarization in the penumbra upon various treatments at 24 h post‐MCAO, and quantitative analysis of CD206/CD86 ratio. Scale bars: 50 µm. F) GSEA enrichment plots between CeO_2_@CO@CM treatment and saline treatment at 24 h post‐MCAO revealing in vivo neuroprotective role of CeO_2_@CO@CM in inhibiting neuroinflammation and neuronal cell death. Each bubble representing a pathway, its horizontal position indicating the normalized enrichment score (NES; NES > 0 enriched, NES < 0 depleted), bubble size proportional to the number of leading‐edge genes, and color intensity reflecting –log_10_(*p* value). G,H) Representative immunofluorescence staining of BrdU (red)/DCX (green)/DAPI (blue) in SVZ upon various treatments at 14 days post‐MCAO reflecting the proliferating neural precursor cells, and quantitative analysis of newly divided neural precursor cells. Scale bars: 50 µm. I,J) Representative immunofluorescence staining of BrdU (red)/NeuN (green)/DAPI (blue) upon various treatments in the penumbra at 14 d post‐MCAO reflecting proliferating neurons, and quantitative analysis of newly divided neurons. Scale bars: 50 µm. K) GSEA enrichment plots between CeO_2_@CO@CM treatment and saline treatment at 14 d post‐MCAO revealing in vivo neurogenesis‐promoting role of CeO_2_@CO@CM in promoting neural regeneration and inhibiting glial scarring. Each bubble representing a pathway, its horizontal position indicating the normalized enrichment score (NES; NES > 0 enriched, NES < 0 depleted), bubble size proportional to the number of leading‐edge genes, and color intensity reflecting –log_10_(*p* value). Data were presented as mean ± SEM. Statistical comparisons between two groups were performed using an unpaired two‐tailed Student's *t*‐test, and one‐way ANOVA test was applied for comparisons among multiple groups. ns: not significant; **P < 0.01, ***P < 0.001.

Excessive oxidative stress/neuroinflammation and damaged BBB were the typical pathological features of cerebral ischemia‐reperfusion injury, which also became the essential factors to exacerbate brain injury. Therefore, we mainly investigated in vivo neuroprotective effects of CeO_2_@CO@CM on these pathological factors at 24 h post‐MCAO. First, we investigated the inhibitory effect on oxidative stress and neuroinflammation in vivo. By immunofluorescence staining and ELISA assays, we found that the occurrence of cerebral ischemia‐reperfusion caused a significant oxidative damage to neuronal DNA/RNA, because the 8‐hydroxy‐2′‐deoxyguanosine (8‐OHG) was upregulated and distributed in neurons (**Figure** **6**A,B and S13, Supporting Information). Besides, this induced microglia polarization toward M1 subtype reflected by CD206/CD86 ratio downregulation (Figure 6C–E), and generated a pro‐inflammatory microenvironment with increasing pro‐inflammatory cytokines levels (IL‐1β, IL‐6 and TNF‐α) (Figures S14–S16, Supporting Information). Upon various drug treatment, we found that CeO_2_@CO@CM showed the strongest inhibitory effects on oxidative stress and neuroinflammation, via alleviating oxidative damage to neurons, converting microglia polarization to anti‐inflammatory M2 subtype, downregulating M1‐type cytokines levels (IL‐1β, IL‐6, and TNF‐α), and upregulating M2‐type cytokines levels (IL‐10 and TGF‐β1) (Figure 6C–E; Figures S14–S18, Supporting Information). To further validate neuroprotective effects of CeO_2_@CO@CM, transcriptomics and proteomics assays were performed at 24 h post‐MCAO. In Figure 6F, GSEA enrichment plots of RNA‐sequencing revealed that CeO_2_@CO@CM could significantly inhibit neuroinflammation and neuronal cell death via downregulating multiple pathways, i.e., positive regulation of cytokine production involved in inflammatory response, regulation of microglial cell activation, positive regulation of reactive oxygen species metabolic process, positive regulation of apoptotic signaling pathway, positive regulation of neuron death. From another viewpoint, Gene Ontology (GO) functional enrichment analysis of protein‐sequencing also confirmed the in vivo neuroprotective effects of CeO_2_@CO@CM (Figure S19, Supporting Information).

Given that BBB disruption is also a crucial contributor to the aggravation of brain damage, we continued to performed Evans blue extravasation in the brain to examine in vivo BBB‐protection capability of CeO_2_@CO@CM. As expected, the cerebral ischemia‐reperfusion led to a severe BBB damage, reflected by the markedly increased Evans blue extravasation into ischemic hemisphere (Figure S20, Supporting Information). Upon various drug treatments, the Evans blue extravasation was inhibited at 24 h with varying degrees, where CeO_2_@CO@CM exhibited the strongest inhibition effects in comparison with CeO_2_@CO and CeO_2_ groups. This demonstrated that BBB damage caused by the cerebral ischemia‐reperfusion could be effectively alleviated by CeO_2_@CO@CM. These benefits were primarily owing to its multiple design advantages, including the targeted delivery, controlled CO release, and ROS scavenging.

### 2.7 In Vivo Promotion Effect on Neurogenesis

To validate in vivo promotion effect of CeO_2_@CO@CM on neurogenesis, the immunofluorescence double‐staining was further conducted after cerebral ischemia‐reperfusion, including 5‐bromodeoxyuridinc (BrdU)/doublecortin (DCX) to track proliferating neural precursor cells in SVZ, and BrdU/NeuN to track proliferating neurons in the penumbra. In Figure 6G–J and Figures S21 and S22 (Supporting Information), it was found that the increased fluorescence signals in CeO_2_@CO@CM and CeO_2_@CO treatment group, confirming that the proliferation of neuronal precursors and mature neurons were facilitated attributing to the controlled CO release. By comparison, the CeO_2_@CO@CM treatment had stronger promotion effects on neurogenesis than CeO_2_@CO treatment benefiting from the enhanced targeted delivery potentials. The number of newly divided neural precursor cells in CeO_2_@CO@CM treatment was ≈5.9‐fold than that of saline group, while the number of newly divided neurons in CeO_2_@CO@CM treatment was ≈7.7‐fold than that of saline group. We further conducted transcriptomics and proteomics assays. As shown in Figure 6K, GSEA enrichment plots of RNA‐sequencing showed that CeO_2_@CO@CM not only promoted neural regeneration via upregulating several pathways (i.e., positive regulation of axonogenesis, positive regulation of synapse assembly, central nervous system neuron development), but also inhibited glial scarring via downregulating multiple pathways (i.e., positive regulation of gliogenesis, astrocyte activation, glial cell proliferation). In addition, GO functional enrichment analysis of protein‐sequencing validated the promotion effect on neurogenesis of CeO_2_@CO@CM (Figure S23, Supporting Information). Taken together, these evidences fully demonstrated the capability of CeO_2_@CO@CM in facilitating the endogenous neurogenesis in a mouse MCAO model. Therefore, we rationally considered that this molecular design of CeO_2_@CO@CM improved CO therapy in a safe and controllable manner, which was hoped to accelerate brain remodeling.

### 2.8 Dual‐Channel Therapeutic Effects on Reducing Brain Injury and Restoring Neurofunctions

**Figure 7:**
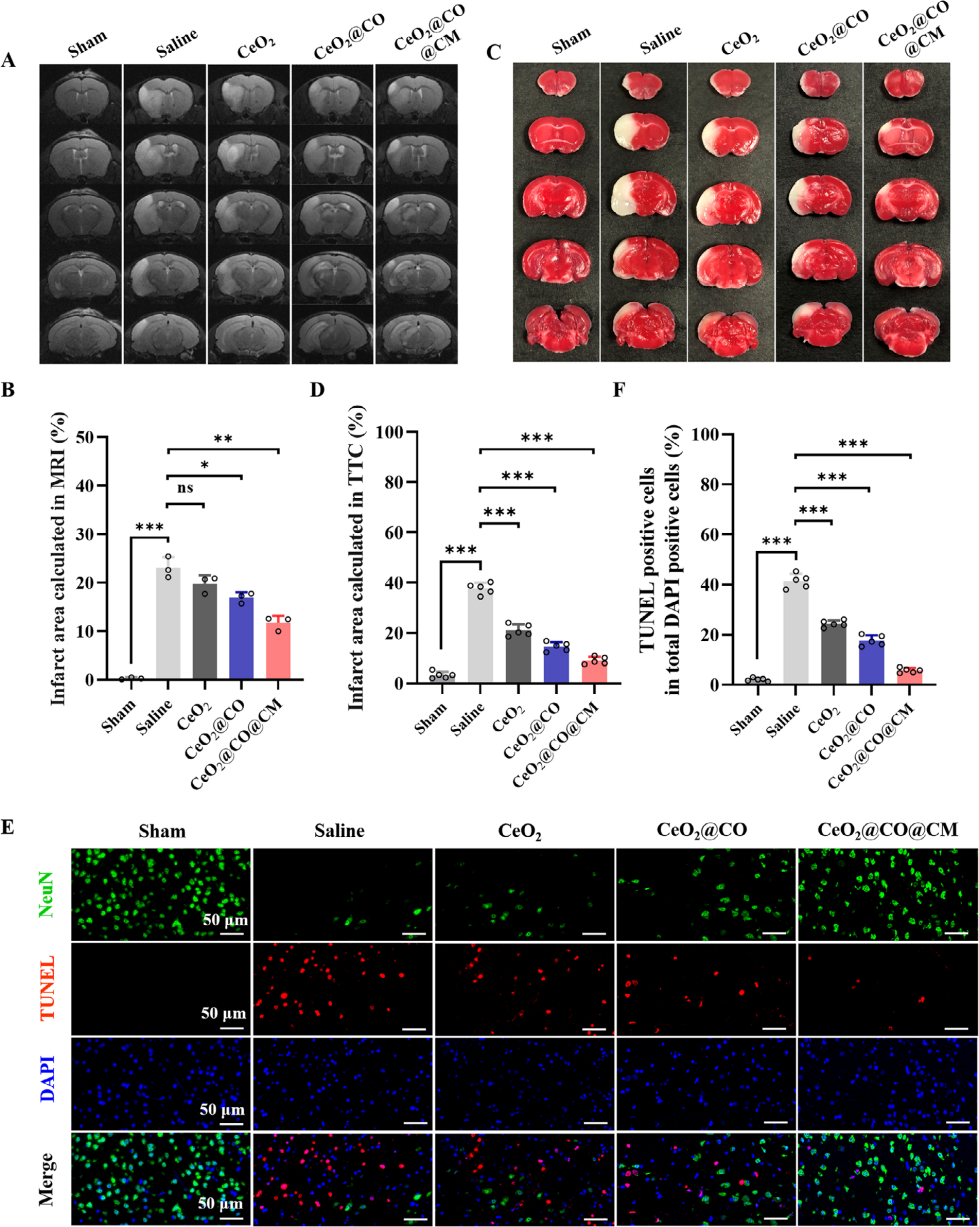
Dual‐channel therapeutic effects on reducing brain injury. A,B) Representative T2W magnetic resonance images of the brains upon various treatments at 4 d post‐MCAO, and quantitative analysis of infarct area. C,D) Representative TTC staining images of the brains upon various treatments at 4 d post‐MCAO (Infarcted regions and healthy brain tissues were stained as white and red), and quantitative analysis of infarct area. E,F) Representative immunofluorescence staining of TUNEL (red)/NeuN (green)/DAPI (blue) reflecting neuronal apoptosis in the penumbra upon various treatments at 4 d post‐MCAO, and quantitative analysis of TUNEL positive cells. Scale bars: 50 µm. Data were presented as mean ± SEM. Statistical comparisons between two groups were performed using an unpaired two‐tailed Student's *t*‐test, and one‐way ANOVA test was applied for comparisons among multiple groups. ns: not significant; *P < 0.05, **P < 0.01, ***P < 0.001.

To harness the multiple neuroprotective functions of CeO_2_@CO@CM and its ability to enhance neurogenesis, we extended our investigations to evaluate this dual‐channel therapeutic effects on reducing brain injury and promoting neurofunctional recovery. To systematically evaluate brain injury, the magnetic resonance imaging (MRI), a standard diagnostic tool for stroke, was used to identify infarct regions. As shown in **Figure** **7**A,B, T2‐weighted images showed the obvious lesions in MCAO mice, whereas no infarct zones were observed in sham group. Upon various drug treatments, the lesion volumes were reduced to varying extents, with CeO_2_@CO@CM group demonstrating the largest reduction. Besides, complementary analysis using 2,3,5‐triphenyltetrazolium chloride (TTC) corroborated these findings, in which the infarcted regions and healthy brain tissues were stained as white and red, respectively. TTC results indicated large infarct areas in MCAO mice, which were effectively diminished with CeO_2_@CO@CM treatment (Figure 7C,D). These results suggested this biomimetic CO nanogenerator substantially reduced infarction regions and impeded infarct progression. Further, we examined neuronal apoptosis in the penumbra via NeuN/TUNEL staining (Figure 7E,F). This analysis showed that MCAO‐induced neuronal apoptosis was significantly mitigated by CeO_2_@CO@CM, and thereby enhancing neuronal survival and restoring neuronal signaling. Collectively, these results illustrated that this biomimetic CO nanogenerator effectively reduced the brain injury following cerebral ischemia‐reperfusion.

**Figure 8:**
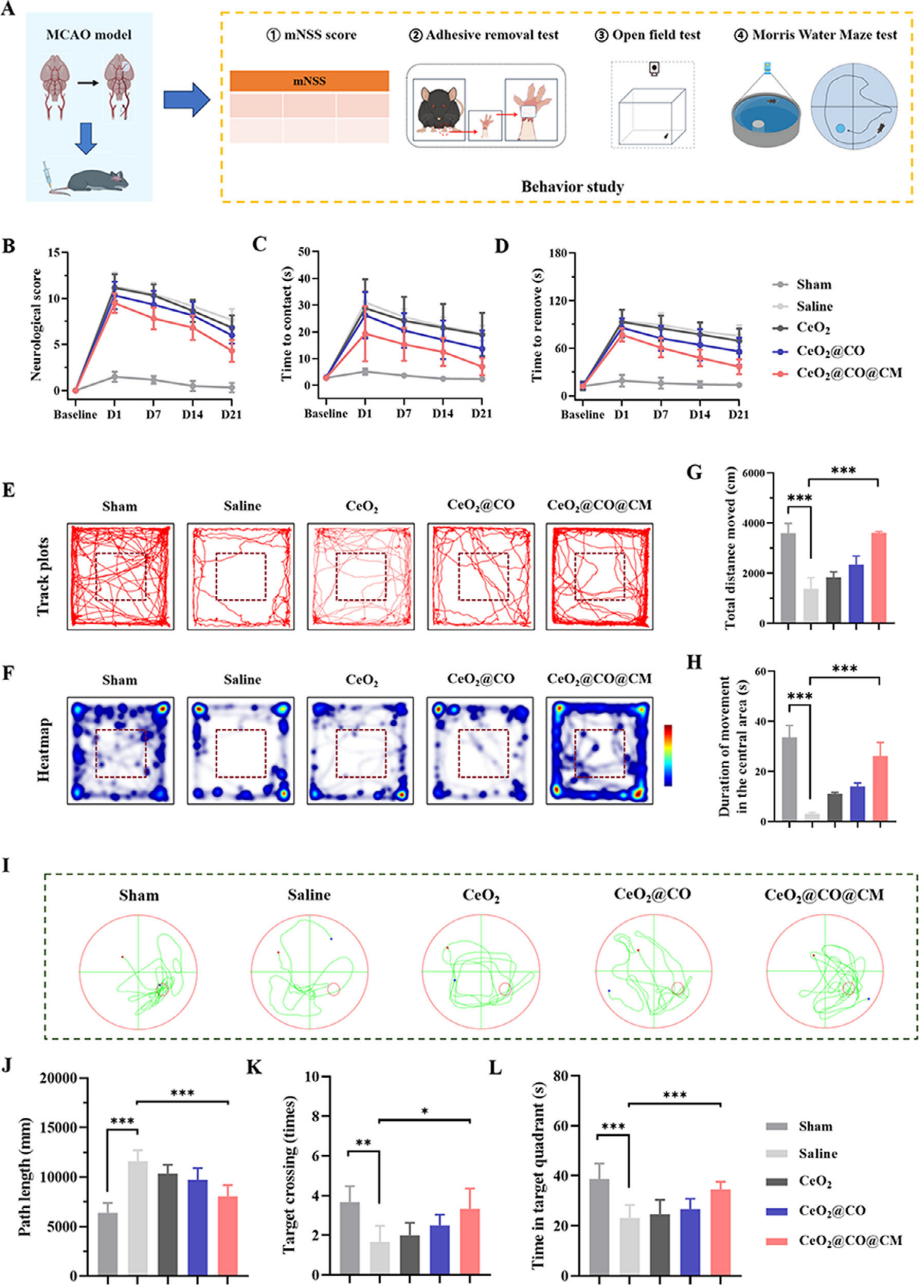
Dual‐channel therapeutic effects on restoring neurofunctions. A) Schematic illustration of the performed behavior tests. B) The assessment of sensorimotor functions using mNSS score at different time points. C) Time of contact in MCAO mice upon various treatments in adhesive removal test. D) Time of remove sticker in MCAO mice upon various treatments in adhesive removal test. E,F) Representative track plots and corresponding heatmaps in open field test. G) Total distance moved in open field test. H) Duration of movement in the central area in open field test. I) The swimming trajectories in MWM test. J) Path length of MCAO mice at day 21 upon various treatments in MWM test. K) Target crossing of MCAO mice at day 21 upon various treatments in MWM test. L) Time in target quadrant of MCAO mice at day 21 upon various treatments in MWM test. Data were presented as mean ± SEM. Statistical comparisons between two groups were performed using an unpaired two‐tailed Student's *t*‐test, and one‐way ANOVA test was applied for comparisons among multiple groups. *P < 0.05, **P < 0.01, ***P < 0.001.

In addition, to assess dual‐channel therapeutic effect on improving neurofunctions, the neurological scoring and behavioral tests were performed, as illustrated in **Figure** **8**A. First, the modified neurologic severity score (mNSS) was conducted to assess sensorimotor impairment, showing that CeO_2_@CO@CM treatment enabled this score to gradually decrease within 21 d and its performance was superior to other treatment groups (Figure 8B). This result illustrated that biomimetic CO nanogenerator overall reversed the sensorimotor deficits induced by cerebral ischemia‐reperfusion. Besides, the capacity of this mouse to integrate sensory and motor functions was assessed using an adhesive removal test. This test required the mice to discern and remove an adhesive sticker affixed to their left forelimb through motor coordination. As shown in Figure 8C,D, prior to the MCAO surgery, all experimental mice had a similar level in the time needed for both contact and removal. However, after MCAO surgery, there was a significant increase in the time required for contact and sticker removal in experimental mice. And the CeO_2_@CO@CM treatment indeed led to a reduction in duration of contact and sticker removal, suggesting that CeO_2_@CO@CM treatment ameliorates the sensory and motor impairments in mice following cerebral ischemia‐reperfusion. Besides, we performed open field test to assess motor function and physical activity upon CeO_2_@CO@CM treatment, where those mice treated with CeO_2_@CO@CM showed an obvious increase in the total distance moved, implying the enhanced locomotor activity and good recovery of physical function compared to other treatments (Figure 8E–G). Also, duration of movement in the central area was used to assess anxiety‐like symptoms (Figure 8H). Result showed that more time was spent in the central area for those mice receiving CeO_2_@CO@CM treatment, indicating reduced anxiety‐like symptoms.

Learning and memory dysfunctions are also common outcomes, and therefore it was further evaluated via a Morris Water Maze (MWM) test. Before MCAO surgery, experimental mice were trained with the similar escape latency (Figure S24, Supporting Information). Then, this test was performed at 21 days after MCAO. The mice treated with CeO_2_@CO@CM showed better recovery of learning and spatial memory functions than other treatments (Figure 8I), as they travelled shorter swimming path (Figure 8J), exhibited the increased crossing numbers (Figure 8K), and stayed for longer duration in the target quadrant (Figure 8L). These data showed that CeO_2_@CO@CM treatment enabled MCAO mice to search for platform with more purposes, indicating that the spatial memory and learning ability were effectively improved. Taken together, the dual‐channel therapeutic strategy based on CeO_2_@CO@CM could improve neurobehavioral functions cerebral ischemia‐reperfusion injury.

### 2.9 In Vivo Biosafety Evaluations

**Figure 9:**
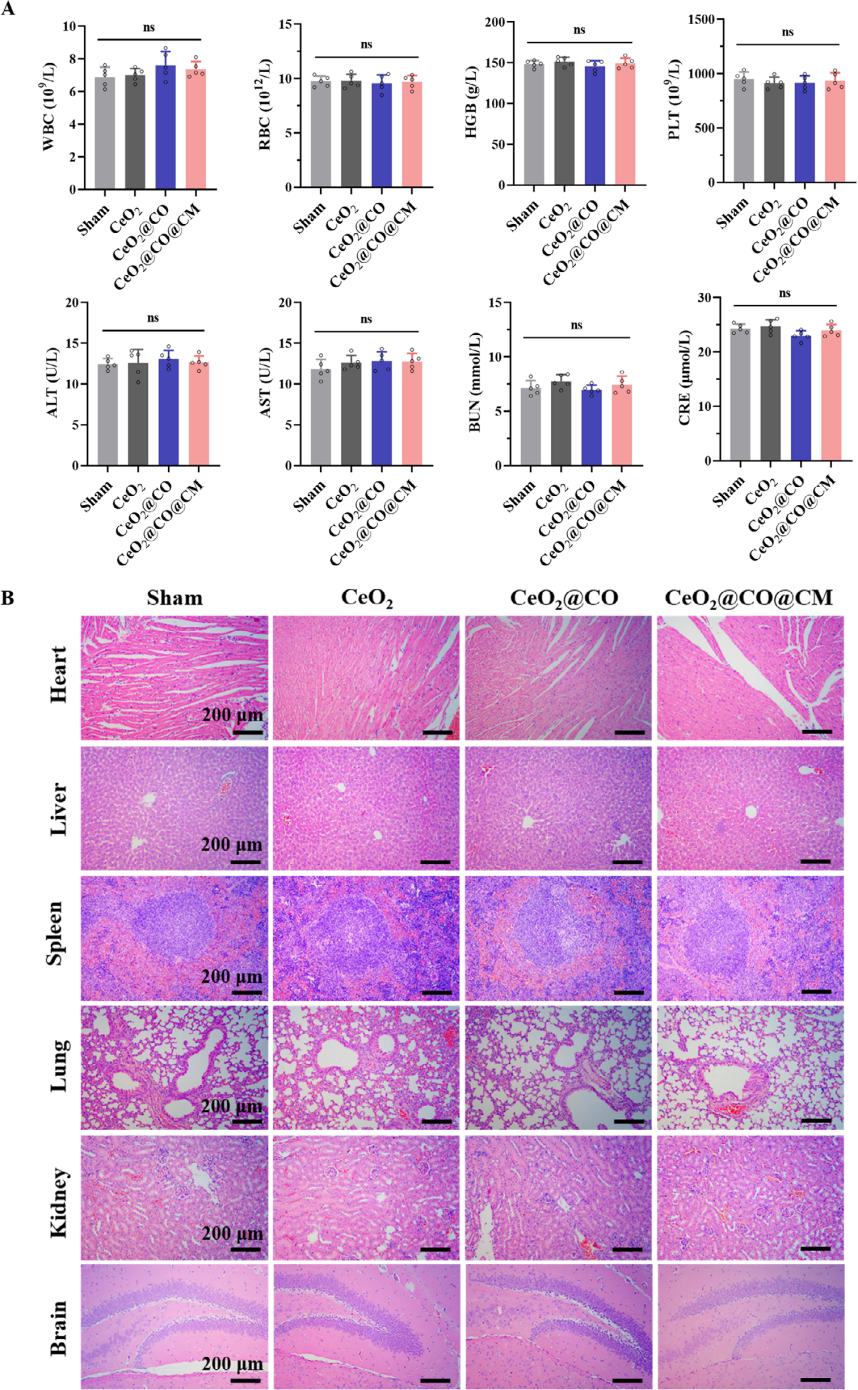
In vivo biosafety assessment of CeO_2_@CO@CM at hematological and organ levels. A) Side effects on blood cells and liver, kidney functions examined by the blood routine and biochemical tests, including WBC, RBC, HGB, PLT, ALT, AST, BUN, CRE. B) Representative H&E staining of major organs, including heart, liver, spleen, lung, kidney, reflecting their injury situation. Scale bars: 200 µm. Data were presented as mean ± SEM. Statistical comparisons between two groups were performed using an unpaired two‐tailed Student's *t*‐test, and one‐way ANOVA test was applied for comparisons among multiple groups. ns: not significant.

Biosafety is a critical prerequisite for therapeutic drugs in clinical applications. To assess the in vivo biosafety of CeO_2_@CO@CM, we conducted the corresponding evaluations at hematological and organ levels. First, routine blood and biochemical tests were performed using blood samples collected from tail vein at day 7. Results showed no significant differences between the CeO_2_@CO@CM and sham groups, in routine hematology markers and several important biochemical markers, i.e., alanine aminotransferase (ALT), aspartate transaminase (AST), blood urea nitrogen (BUN), and creatinine levels (**Figure** **9**A). These findings suggested that the CeO_2_@CO@CM did not adversely affect blood cells, liver and kidney functions. To further investigate potential side effects on major organs, mice were sacrificed at day 7 for the collection of primary organs, including the heart, liver, spleen, lung and kidney. Histopathological analysis using H&E staining revealed no observable damage or morphological changes in these organs upon CeO_2_@CO@CM treatment (Figure 9B). Collectively, these results demonstrated that CeO_2_@CO@CM was biologically safe in vivo.

### 2.10 Discussion

Ischemia‐reperfusion injury in stroke contributes to high mortality rates and long‐term disability. This has posed a significant public health challenge. Unfortunately, the existing pharmaceutical interventions are hardly to modify the progression of ischemia‐reperfusion injury, and only acquire the limited efficacy. Therefore, the advanced pharmacotherapy should be urgently explored to not only minimize brain injury, but also improve long‐term neurological dysfunctions, which remain the biggest obstacle worldwide. The advanced nanotechnology brings a new therapeutic hope. This work focuses on the aspects listed as follows. (1) Traditional neuroprotection strategies have shown the promise in preclinical studies by inhibiting neuronal death in penumbra, but still insufficient to address the long‐term neurological dysfunctions due to the limited self‐repair capacity of the central nervous system. In this work, we find that CO as a neuromodulator, exerts a role in promoting neurogenesis, which is beyond its recognized roles in anti‐inflammation and anti‐oxidation. This reveals a new possibility to address the above challenge via an innovative dual‐channel therapeutic mode of co‐driving neuroprotection and neurogenesis. (2) To implement the effective CO therapy, several challenges still need to be addressed: efficient delivery of CO to ischemic lesion after penetrating BBB; the controlled release of CO at low levels to ensure therapeutic performance and avoid the toxicity risks at high levels. To this end, we develop a biomimetic and ROS‐activated CO nanogenerator, enabling to penetrate BBB, arrive in stroke‐affected regions and release CO in a controlled manner. (3) CO nanomodulator exerts the dual‐channel therapeutic mode via not only reducing neuroinflammation/oxidative stress and inhibiting neuronal apoptosis for exerting multiple neuroprotective effects, but also promoting directed differentiation of neural stem cells towards mature neurons for enhancing neurogenesis. This new therapeutic mode demonstrates the significant effects on reversing the brain injury and improving the neurofunctions in a mouse ischemic stroke model. However, this study also exists limitation. For instance, this therapeutic effect is only demonstrated in the mouse, where the large animal and human experiments are lacking. Besides, the in vivo fluorescence data of CeO_2_@CO@CM in the liver are lacking mainly owing to the severe shielding effect of the thick bones, muscles, and partial fur in the dorsal region of the mice in a prone position.

More importantly, we elucidated the mechanism how CO promoted neurogenesis. In this mechanism, the BMEC‐NSC crosstalk and NO molecule were crucial. Therefore, we would like to clarify why the CO was chosen for the construction of nanomedicine, rather than NO. Although NO can mediate neurogenesis, it also reacts with high level of ROS in lesion to produce the highly toxic peroxynitrite (ONOO^−^) for aggravating the disease progression.^[^ ^36^ ^]^ In addition, the NO is commonly inefficient to address neuroinflammatory events for neuroprotection. By comparison, the CO exhibits the superiority in the dual effects of promoting neuroprotection and neurogenesis. Based on these considerations, CO is rationally selected to fabricate biomimetic and ROS‐activated CO nanogenerator for dual‐channel therapy. Besides, it is observed that IL‐10 and TGF‐β1 levels in BV2 are slightly increased upon OGD/R condition or MCAO condition. Similar results are also reported in other literatures.^[^ ^37^, ^38^ ^]^ This may attribute to spontaneous protective responses. And drug treatments further induce the increase in IL‐10 and TGF‐β1 levels. Herein, it also should be noticed that the increased IL‐10 and TGF‐β1 levels by drug treatments aim to normalize inflammatory microenvironment at the acute phase. After addressing neuroinflammation, the nanomedicines will stop administration avoiding the risk of the abnormal pathological damages probably induced by the continuously increased IL‐10 and TGF‐β1 levels.

## 3 Conclusion

As a proof of concept, this work revealed that gas signaling molecule CO not only acted as a neuroprotective agent but also exerted a modulation effect on endogenous neurogenesis via BMEC‐NSC crosstalk. This inspired us to explore an innovative dual‐channel therapeutic strategy integrating neuroprotection with enhanced neurogenesis to address challenge of long‐term neurological dysfunctions after ischemia‐reperfusion injury in stroke. To this end, we further developed a biomimetic and ROS‐sensitive CO nanogenerator, which could penetrate BBB and distributed in stroke‐affected regions for controlled CO release. In a mouse stroke model, intravenous administration of this CO nanogenerator reduced neuroinflammation/oxidative stress and inhibited neuronal apoptosis to exert multiple neuroprotective effects, as well as promoted the proliferation and differentiation of NSC in SVZ to enhance neurogenesis. Consequently, this dual‐channel therapy reduced brain injury and promoted neurofunctional recovery. Besides, it was assessed that this nanogenerator had a satisfactory safety. This work proposed a new dual‐channel strategy enabled by CO nanogenerator, which provided a promising solution for the long‐term neurological dysfunctions in cerebral ischemia‐reperfusion injury.

## 4 Experimental Section

### Materials

Hollow mesoporous cerium dioxide nanoparticles were purchased from Jiangsu XFNANO Materials Tech Co., Ltd. Carbonyl manganese (MnCO) and methanol were obtained from Macklin Biochemical Technology Co., Ltd. DCFH‐DA, TUNEL apoptosis assay kit, and DAPI were obtained from Meilun Biotechnology Co., Ltd. Dulbecco's modified eagle medium (DMEM), FBS, and CCK‐8 were ordered from Invitrogen Biotechnology Co., Ltd. DPPH and ABTS free radical scavenging capacity assay kit were obtained from Solarbio Science & Technology Co., Ltd. IL‐6 ELISA kit, IL‐1β ELISA kit, TNF‐α ELISA kit, IL‐10 ELISA kit, and TGF‐β1 ELISA kit were obtained from R&D Systems. Primary antibodies for immunofluorescence and immunohistochemistry experiments, mainly including 8‐OHG (ab48508), NeuN (ab177487), CD206 (ab64693), CD86 (ab119857), BrdU (ab6326), and DCX (ab18723), were purchased as indicated. Other chemicals were analytical grade without further purification.

### Preparation of CeO_2_@CO@CM

The MnCO (10 mg) was firstly dissolved in methanol (5 mL) to obtain a yellow solution, and then this solution was added to the methanol solution containing hollow mesoporous cerium dioxide (1 mg mL^−1^, 10 mL) for stirring overnight. Afterward, CeO_2_@CO was collected following centrifugation and washing with double‐distilled water. Then, as‐prepared macrophage cell membrane (1 mg) was dispersed in double‐distilled water (1 mL) at temperature below 20 °C. Next, this solution was mixed with the aqueous solution of CeO_2_@CO (1 mg mL^−1^), and subjected to ultrasound for 5 min at temperature below 20 °C. Finally, this mixture solution was coextruded through a polycarbonate membrane (200 nm) for 10 recycles by a mini‐extruder to obtain CeO_2_@CO@CM.

### Physicochemical Characterizations

Microscopic morphology was observed by high‐resolution transmission electron microscopy; Elemental composition and distribution were detected by X‐ray photoelectron spectroscopy (XPS). The coating effect of macrophage cell membrane was assessed by dye‐labeling technology and CLSM observation. The MnCO loading was evaluated by ICP‐OES. The CO release rate was quantified by fluorescence probe. Z‐average diameter and zeta potential were monitored by Zetasizer Nano ZS. ROS scavenging property was examined by EPR, DPPH, and ABTS assays.

### Cell Culture and Cell Model of OGD/R

Brain endothelial cells (Bend.3), microglial cells (BV2), hippocampal neuronal cells (HT22), and neural stem cells were obtained from Cell Bank of Typical Culture Collection of Chinese Academy of Sciences for the cell culture in a 5% CO_2_ incubator at 37 °C. To establish the cell model of OGD/R, cells were firstly cultured in a glucose‐free DMEM and placed in a hypoxia incubator. Then, cells were transferred to a 5% CO_2_ incubator with normal oxygen supply.

### In Vitro Evaluation of CO Release

To assess the CO release rate, CeO_2_@CO@CM solution was mixed with different concentrations of H_2_O_2_ (0, 0.1, 0.5, 1.0 × 10^−3^ m). Then, CO fluorescence probe (10 × 10^−6^ m) and PbCl_2_ (10 × 10^−6^ m) were added to the above solution. At different time points, the fluorescence spectrum was examined at 510 nm to quantify the released CO amount.

For intracellular CO release, HT22 cells with or without OGD/R condition were co‐cultured with different drugs for 12 h, and then the medium was replaced with fresh medium containing CO fluorescence probe (2 × 10^−6^ m) and PdCl_2_ (2 × 10^−6^ m). After incubation for another 30 min, these cells were washed with PBS and observed by fluorescence microscope.

### In Vitro Evaluation of ROS Regulation

DCFH‐DA assay kit was used to detect intracellular ROS level for in vitro evaluation of ROS regulation. The HT22 cells with or without OGD/R condition were incubated with different concentrations of drugs for 24 h, and then DCFH‐DA was added for 30 min. Subsequently, cells were washed with PBS three times. The resulting cells were trypsinized and collected in PBS for fluorescence microscope observation (green channel) and analysis of MFI by Image J.

### In Vitro Evaluations of Neuroprotective Effects

The HT22 cells with or without OGD/R condition were incubated with different concentrations of drugs for 24 h, and then culture medium was replaced with fresh medium containing CCK‐8 agent and continued to incubate for 2 h. Finally, absorbance value was measured at 450 nm via a microplate reader to reflect cell viability for in vitro evaluation of neuroprotective role.

### In Vitro Evaluations of Regulating Microglial Polarization and Neuroinflammation

To assess the effect of CeO_2_@CO@CM on microglial polarization in vitro, the BV‐2 cells were subjected to OGD/R and then various drugs were added to medium. After 24 h, the immunofluorescence staining of CD86 and CD206 was conducted to examine microglial polarization. Besides, ELISA assays were performed to quantify the level of cytokines (TNF‐α, IL‐6, IL‐1β, IL‐10, TGF‐β1).

### In Vitro Evaluations of Regulating the Proliferation and Differentiation of NSC

The brain endothelial cells with OGD/R condition were treated with various CO‐releasing drugs for 24 h, and then culture medium was collected as ECCM. The NSC with or without OGD/R condition were treated with ECCM for the support of CCK‐8 assay to assess cell counts and proliferation situation of NSC, as well as RT‐PCR, cell immunostaining and western blot assays to examine expressions of relevant biomarkers and differentiation situation of NSC.

### In Vitro BBB Penetrating Assay

In vitro BBB model was established by seeding Bend.3 cells in upper insert of a transwell plate and incubating for one week until a transendothelial electrical resistance of over 250 Ω cm^2^ was achieved. HT22 cells were subsequently seeded in the lower chambers and incubated for 24 h. Cy5.5‐labeled drugs were added to the upper chambers and incubated for 12 h. The HT22 cells in the lower chambers were visualized using CLSM after DAPI staining.

### Animal Model of MCAO and Therapy Strategy

The MCAO model was constructed using a suture‐occluded method. Male C57/BL6 mice (25–30 g) were anesthetized with 1% pentobarbital. Left common carotid artery, external carotid artery, and internal carotid artery were carefully isolated. A nylon suture was then inserted from the external carotid artery to the internal carotid artery to block the blood supply to middle cerebral artery. After 90 min, the suture was withdrawn to allow reperfusion. The sham group underwent surgery without occlusion. Animals were divided into the following groups: Sham, Saline, CeO_2_, CeO_2_@CO, CeO_2_@CO@CM. The MCAO model was performed on the latter four groups. Mice were maintained under a natural circadian rhythm with ad libitum access to food and water prior to surgery. This animal experiment was conducted in accordance with the Animal Protection Guidelines of Fujian Medical University (IACUC FJMU 2022‐0608) and conformed to the “Guide for the Protection and Use of Experimental Animals” set forth by the American National Institutes of Health.

For drug administration, each group (Saline, CeO_2_, CeO_2_@CO, CeO_2_@CO@CM) received corresponding drug injections at a dosage of 2 mg kg^−1^ via the tail vein after 1 h of reperfusion at day 0, and day 1 and day 2 post‐MCAO. The sham group followed the same protocol but did not receive drug treatment.

### In Vivo and Ex Vivo Evaluations of Biodistribution

Cy5.5‐labeled drugs were administered to MCAO mice via the tail vein. At various time points, the mice were imaged using an IVIS fluorescence imaging system. In addition, major organs (heart, liver, spleen, lung, kidney, brain) were collected post‐sacrifice for ex vivo fluorescence imaging to monitor the biodistribution of CeO_2_@CO@CM in the mice.

### In Vivo Evaluations of Neuroprotection and Neurogenesis‐Enhancing Effects

To assess the neuroprotection and neurogenesis‐enhancing effects, we conducted immunofluorescence staining, ELISA assay, and Evans blue staining. For immunofluorescence staining, mice were transcardially perfused with saline followed by 4% paraformaldehyde (PFA). Brain sections were collected and fixed in 4% PFA. Sections with ≈25 µm thick were prepared, treated with Triton X‐100 for permeabilization, and serum treatment for overnight incubation with primary antibodies, including anti‐CD206, anti‐CD86, anti‐8‐OHG, anti‐NeuN, anti‐BrdU, and anti‐DCX. After washing three times with PBS, sections were incubated with Alexa Fluor‐conjugated secondary antibodies, and DAPI was used for nuclear staining. For the ELISA assay, the ischemic brain tissues were collected to measure pro‐inflammatory cytokine levels (TNF‐α, IL‐6, IL‐1β, IL‐10, TGF‐β1) according to the respective kits’ instructions. The Evans blue staining involved intravenous injection of 2% Evans blue solution, followed by transcardial perfusion with saline after 1.5 h.

### Dual‐Channel Therapeutic Effects on Reducing Brain Injury and Restoring Neurofunctions

To assess the dual‐channel therapeutic effect on reducing brain injury, MRI and TTC staining were performed to evaluate infarct volume in the brain. At the pre‐determined time point, T2‐weighted imaging of the brain tissues was conducted using a 7.0 T MRI system at day 4 post‐MCAO, with further analysis carried out using accompanying software. For TTC staining, the mice were euthanized at day 4 post‐MCAO, and their brains were extracted for analysis. The brains were sliced into ≈2‐mm‐thick sections along the coronal plane and stained with 2% TTC at 45 °C for 10 min. The infarct volume was quantified using ImageJ software.

To evaluate the dual‐channel therapeutic effect on restoring neurofunctions, the authors conducted behavioral assessments, including the mNSS, the adhesive removal test, and the Morris water maze test. The mNSS assessed sensorimotor functions through a blinded scoring method, with scores ranging from 0 to 18, where lower scores indicated better neurofunctional recovery. All assessments were performed daily at the same time by the same experimenter, who was blinded to the experimental conditions.

The adhesive removal test was implemented to assess sensory and motor functions. A piece of adhesive tape was affixed to the distal‐radial region of each forelimb. The mice were then placed in a transparent plexiglass cage devoid of bedding to facilitate observation. Prior to MCAO, each mouse underwent a training period of three days, during which they were tested five times daily to familiarize them with the procedure. Following MCAO, five trials were conducted each day. Each trial ended either when the adhesive tape was removed from the left forelimb or after 180 s had elapsed.

Open field test was used to evaluate the locomotor activity. Mice were introduced into a 40 cm high, opaque acrylic enclosure measuring 40 cm × 40 cm and given 10 min for exploration. A central 20 cm × 20 cm square defined the arena's inner zone. An overhead camera recorded behavior, subsequently analyzed by ANY‐maze software. To maintain environmental consistency between subjects, the arena was thoroughly cleaned with 75% ethanol after each session to eliminate odors and waste. Testing resumed only after the ethanol had fully evaporated.

To evaluate learning and memory, the Morris water maze experiment was performed, where the water temperature was maintained at 24 °C. In the training stage, mice were randomly placed into the water from designated quadrants and allowed to swim freely until they located a submerged resting platform, which was hidden 1 cm below the water surface. The escape latency was recorded, with a maximum swimming time of 90 s. If a mouse failed to find the platform within this time, it was gently guided to the platform with a stick and allowed to remain there for 15 s. Upon starting test, the platform was removed, and the mice were allowed to explore randomly to locate the area where the platform had been. The swimming path length, target crossing, and time in target quadrant were recorded.

### Biosafety Assessment

The biosafety of CeO_2_@CO@CM was first evaluated using a CCK‐8 assay on common brain cell types, including Bend.3, BV2, and HT22 cells. Cells were seeded in a 96‐well plate and cultured for 24 h in complete DMEM. They were then treated with varying concentrations of drugs for 24 h, with the PBS‐treated cells serving as a negative control. Following treatment, culture medium was replaced with fresh medium containing CCK‐8 reagent, and incubation continued for an additional 2 h. Absorbance was measured at 450 nm using a microplate reader to assess cell viability.

Subsequently, blood was collected from inner canthus at day 7 for routine blood analysis and biochemical tests to evaluate physiological indices, as well as liver and kidney functions. Finally, primary organs were harvested for H&E staining at day 7 to assess potential organ injury.

### RNA Sequencing Analysis

Isolated brain tissues were collected sent to Novogene Co., Ltd. (Beijing, China) for RNA‐sequencing library preparation. Transcriptome sequencing was performed by DNBSEQ‐T7 platform. Data quality was controlled by FastQC tool after removing adaptor sequences, ambiguous “N” nucleotides (proportion of “N” > 5%), and low‐quality sequences (quality score < 10). The ggplotR package was used to identify StringTie genes. Differences were statistically significant for |log_2_ fold change| ≥ 1 or *P* < 0.05.

### Statistical Analysis

Statistical results were analyzed through unpaired Student's *t*‐test (two‐tailed) for comparison between unpaired two‐groups, and one‐way analysis of variance (ANOVA) test for multigroup data comparison. The form of mean ± SEM was used to show data, with statistical significance at ^*^P < 0.05, ^**^P < 0.01, ^***^P < 0.001.

### Ethical Issues on Animal Experiments

The animal experiments were conducted according to Animal Protection Guidelines of Fujian Medical University (IACUC FJMU 2022‐0608), which were also conformed to the “Guide for the Protection and Use of Experimental Animals” of American National Institutes of Health.

## Conflict of Interest

The authors declare no conflict of interest.

## Author Contributions

X.N. and B.G. contributed equally to this work. G.Z., B.G., and D.K. proposed and supervised project. X.N. and B.G. performed physicochemical characterizations. X.N., B.G., Y.Z., Q.L., C.Z., Y.D., J.X., M.L., Y.Z., and Y.W. performed in vitro and in vivo intervention effects and biosafety. X.N., B.G., H.H., and Z.L. performed intervention mechanism experiments. G.Z., B.G., D.K., X.N., Y.L., Y.L., Y.S., and P.W. contributed to discussions about experimental results. X.N. and B.G. wrote this manuscript and Supporting Information. All authors have given approval to the final version of the manuscript.

## Supporting information



Supporting Information

## Data Availability

The data that support the findings of this study are available from the corresponding author upon reasonable request.
