# Supplementary material for: Carbon Monoxide Nanomodulator Reverses Ischemia‐Reperfusion Injury in Stroke: A Novel Dual‐Channel Therapy Mode of Co‐driving Neuroprotection and Neurogenesis
Source: Adv Sci (Weinh). 2025 Oct 17;13(1):e12333. doi: 10.1002/advs.202512333 (PMC12766992; doi:10.1002/advs.202512333)
Supplement: Supplementary file 1 — Supporting Information [file ADVS-13-e12333-s001.docx]

Supplementary Information for

**Carbon monoxide nanomodulator reverses ischemia-reperfusion injury in stroke: a novel dual-channel therapy mode of co-driving neuroprotection and neurogenesis**

**MATERIALS AND METHODS**

**Preparation of CeO_2_@CO@CM**

The MnCO (10 mg) was firstly dissolved in methanol (5 mL) to obtain a yellow solution, and then this solution was added to the methanol solution containing hollow mesoporous cerium dioxide (1 mg/mL, 10 mL) for stirring overnight. Afterwards, CeO_2_@CO was collected following centrifugation and washing with double-distilled water.

Then, the as-prepared macrophage cell membrane (1 mg) was dispersed in double-distilled water (1 mL) at temperature below 20 ℃. Next, this solution was mixed with the aqueous solution of CeO_2_@CO (1 mg/mL), and subjected to ultrasound for 5 min at temperature below 20 ℃. Finally, this mixture solution was coextruded through a polycarbonate membrane (200 nm) for 10 recycles by a mini-extruder to obtain CeO_2_@CO@CM.

**Physicochemical characterizations**

Microscopic morphology was observed by high-resolution transmission electron microscopy; Elemental composition and distribution were detected by X-ray photoelectron spectroscopy (XPS); The coating effect of macrophage cell membrane was assessed by dye-labeling technology and CLSM observation; The MnCO loading was evaluated by ICP-OES; The CO release rate was quantified by fluorescence probe; Z-average diameter and zeta potential at different conditions were monitored by Zetasizer Nano ZS. ROS scavenging property was examined by EPR, DPPH and ABTS assays.

**Cell culture and cell model of OGD/R**

Brain endothelial cells (Bend.3), microglial cells (BV2), hippocampal neuronal cells (HT22), and neural stem cells were obtained from Cell Bank of Typical Culture Collection of Chinese Academy of Sciences for the cell culture in a 5% CO_2_ incubator at 37 °C. To establish the cell model of OGD/R, cells were firstly cultured in a glucose-free DMEM and placed in a hypoxia incubator. Then, cells were transferred to a 5% CO_2_ incubator with normal oxygen supply.

***In vitro* evaluation of CO release**

To assess the CO release rate, CeO_2_@CO@CM solution was mixed with different concentrations of H_2_O_2_ (0, 0.1, 0.5, 1.0 mM). Then, CO fluorescence probe (10 μM) and PbCl_2_ (10 μM) were added to the above solution. At different time points, the fluorescence spectrum was examined at 510 nm to quantify the released CO amount.

For intracellular CO release, HT22 cells with or without OGD/R condition were co-cultured with different drugs for 12 h, and then the medium was replaced with fresh medium containing CO fluorescence probe (2 μM) and PdCl_2_ (2 μM). After incubation for another 30 min, these cells were washed with PBS and observed by fluorescence microscope.

***In vitro* evaluation of ROS regulation**

DCFH-DA assay kit was used to detect intracellular ROS level for *in vitro* evaluation of ROS regulation. The HT22 cells with or without OGD/R condition were incubated with different concentrations of drugs for 24 h, and then DCFH-DA was added for 30 min. Subsequently, cells were washed with PBS three times. The resulting cells were trypsinized and collected in PBS for fluorescence microscope observation (green channel) and analysis of mean fluorescence intensity by Image J.

***In vitro* evaluations of neuroprotective effects**

The HT22 cells with or without OGD/R condition were incubated with different concentrations of drugs for 24 h, and then culture medium was replaced with fresh medium containing CCK-8 agent and continued to incubate for 2 h. Finally, absorbance value was measured at 450 nm *via* a microplate reader to reflect cell viability for *in vitro* evaluation of neuroprotective role.

***In vitro* evaluations of regulating microglial polarization and neuroinflammation**

To assess the effect of CeO_2_@CO@CM on microglial polarization *in vitro*, the BV-2 cells were subjected to OGD/R and then various drugs were added to medium. After 24 h, the immunofluorescence staining of CD86 and CD206 was conducted to examine microglial polarization. Besides, ELISA assays were performed to quantify the level of cytokines (TNF-α, IL-6, IL-1β, IL-10, TGF-β1).

***In vitro* evaluations of regulating the proliferation and differentiation of NSC**

The brain endothelial cells with OGD/R condition were treated with various CO-releasing drugs for 24 h, and then culture medium was collected as ECCM. The NSCs with or without OGD/R condition were treated with ECCM for the support of CCK-8 assay to assess cell counts and proliferation situation of NSC, as well as RT-PCR, cell immunostaining and western blot assays to examine expressions of relevant biomarkers and differentiation situation of NSC.

***In vitro* BBB penetrating assay.**

*In vitro* blood-brain barrier (BBB) model was established by seeding Bend.3 cells in upper insert of a transwell plate and incubating for one week until a transendothelial electrical resistance of over 250 Ω·cm² was achieved. HT22 cells were subsequently seeded in the lower chambers and incubated for 24 h. Cy5.5-labeled drugs were added to the upper chambers and incubated for 12 h. The HT22 cells in the lower chambers were visualized using CLSM after DAPI staining.

**Animal model of MCAO and therapy strategy**

The middle cerebral artery occlusion (MCAO) model was constructed using a suture-occluded method. Male C57/BL6 mice (25-30 g) were anesthetized with 1% pentobarbital. Left common carotid artery, external carotid artery, and internal carotid artery were carefully isolated. A nylon suture was then inserted from the external carotid artery to the internal carotid artery to block the blood supply to middle cerebral artery. After 90 min, the suture was withdrawn to allow reperfusion. The sham group underwent surgery without occlusion. Animals were divided into the following groups: Sham, Saline, CeO_2_, CeO_2_@CO, CeO_2_@CO@CM. The MCAO model was performed on the latter four groups. Mice were maintained under a natural circadian rhythm with ad libitum access to food and water prior to surgery. This animal experiment was conducted in accordance with the Animal Protection Guidelines of Fujian Medical University (IACUC FJMU 2022-0608) and conformed to the “Guide for the Protection and Use of Experimental Animals” set forth by the American National Institutes of Health.

For drug administration, each group (Saline, CeO_2_, CeO_2_@CO, CeO_2_@CO@CM) received corresponding drug injections at a dosage of 2 mg/kg via the tail vein after 1 h of reperfusion at day 0, and day 1 and day 2 post-MCAO. The sham group followed the same protocol but did not receive drug treatment.

***In vivo* and *ex vivo* evaluations of biodistribution**

Cy5.5-labeled drugs were administered to MCAO mice via the tail vein. At various time points, the mice were imaged using an IVIS fluorescence imaging system. Additionally, major organs (heart, liver, spleen, lung, kidney, brain) were collected post-sacrifice for *ex vivo* fluorescence imaging to monitor the biodistribution of CeO_2_@CO@CM in the mice.

***In vivo* evaluations of neuroprotection and neurogenesis-enhancing effects**

To assess the neuroprotection and neurogenesis-enhancing effects, we conducted immunofluorescence staining, ELISA assay, and Evans blue staining. For immunofluorescence staining, mice were transcardially perfused with saline followed by 4% paraformaldehyde (PFA). Brain sections were collected and fixed in 4% PFA. Sections with ~ 25μm thick were prepared, treated with Triton X-100 for permeabilization, and serum treatment for overnight incubation with primary antibodies, including anti-CD206, anti-CD86, anti-8-OHG, anti-NeuN, anti-BrdU, and anti-DCX. After washing three times with PBS, sections were incubated with Alexa Fluor-conjugated secondary antibodies, and DAPI was used for nuclear staining. For the ELISA assay, the ischemic brain tissues were collected to measure the cytokine levels (TNF-α, IL-6, IL-1β, IL-10, TGF-β1) according to the respective kits' instructions. The Evans blue staining involved intravenous injection of 2% Evans blue solution, followed by transcardial perfusion with saline after 1.5 h.

**Dual-channel therapeutic effects on reducing brain injury and restoring neurofunctions**
 To assess the dual-channel therapeutic effect on reducing brain injury, Magnetic Resonance Imaging (MRI) and TTC (2,3,5-triphenyltetrazolium chloride) staining were performed to evaluate infarct volume in the brain. At the pre-determined time point, T2-weighted imaging of the brain tissues was conducted using a 7.0 T MRI system at day 4 post-MCAO, with further analysis carried out using accompanying software. For TTC staining, the mice were euthanized at day 4 post-MCAO, and their brains were extracted for analysis. The brains were sliced into ~ 2 mm thick sections along the coronal plane and stained with 2% TTC at 45 °C for 10 minutes. The infarct volume was quantified using ImageJ software.
 To evaluate the dual-channel therapeutic effect on restoring neurofunctions, we conducted behavioral assessments, including the modified neurologic severity score (mNSS), the adhesive removal test, and the Morris water maze test. The mNSS assessed sensorimotor functions through a blinded scoring method, with scores ranging from 0 to 18, where lower scores indicated better neurofunctional recovery. All assessments were performed daily at the same time by the same experimenter, who was blinded to the experimental conditions.

The adhesive removal test was implemented to assess sensory and motor functions. A piece of adhesive tape was affixed to the distal-radial region of each forelimb. The mice were then placed in a transparent plexiglass cage devoid of bedding to facilitate observation. Prior to middle cerebral artery occlusion (MCAO), each mouse underwent a training period of three days, during which they were tested five times daily to familiarize them with the procedure. Following MCAO, five trials were conducted each day. Each trial ended either when the adhesive tape was removed from the left forelimb or after 180 seconds had elapsed.

Open field test was used to evaluate the locomotor activity. Mice were introduced into a 40 cm high, opaque acrylic enclosure measuring 40 cm by 40 cm and given 10 minutes for exploration. A central 20 cm × 20 cm square defined the arena's inner zone. An overhead camera recorded behavior, subsequently analyzed by ANY-maze software. To maintain environmental consistency between subjects, the arena was thoroughly cleaned with 75% ethanol after each session to eliminate odors and waste. Testing resumed only after the ethanol had fully evaporated.
 To evaluate learning and memory, the Morris water maze experiment was performed, where the water temperature was maintained at 24 °C. In the training stage, mice were randomly placed into the water from designated quadrants and allowed to swim freely until they located a submerged resting platform, which was hidden 1 cm below the water surface. The escape latency was recorded, with a maximum swimming time of 90 s. If a mouse failed to find the platform within this time, it was gently guided to the platform with a stick and allowed to remain there for 15 seconds. Upon starting test, the platform was removed, and the mice were allowed to explore randomly to locate the area where the platform had been. The swimming path length, target crossing, and time in target quadrant were recorded.

**Biosafety Assessment**

The biosafety of CeO_2_@CO@CM was first evaluated using a CCK-8 assay on common brain cell types, including Bend.3, BV2, and HT22 cells. Cells were seeded in a 96-well plate and cultured for 24 h in complete DMEM. They were then treated with varying concentrations of drugs for 24 h, with the PBS-treated cells serving as a negative control. Following treatment, culture medium was replaced with fresh medium containing CCK-8 reagent, and incubation continued for an additional 2 h. Absorbance was measured at 450 nm using a microplate reader to assess cell viability.

Subsequently, blood was collected from inner canthus at day 7 for routine blood analysis and biochemical tests to evaluate physiological indices, as well as liver and kidney functions. Finally, primary organs were harvested for H&E staining at day 7 to assess potential organ injury.


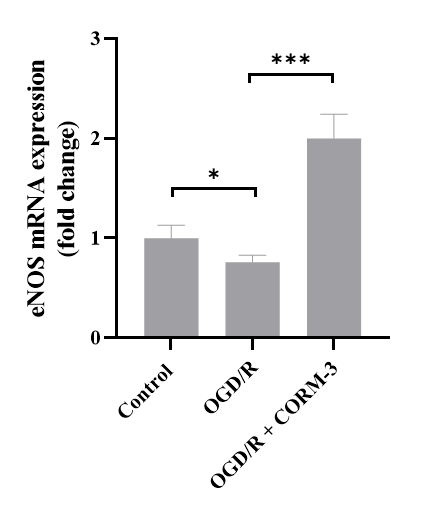


**Figure S1.** eNOS mRNA expression in BMEC upon various treatments.


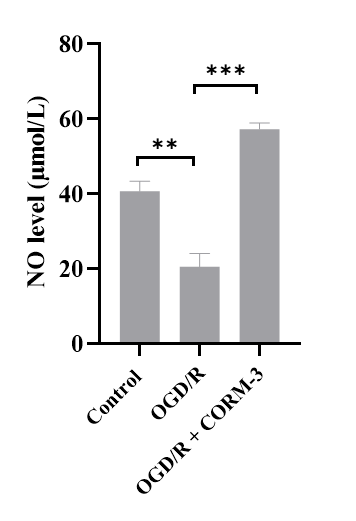


**Figure S2.** The produced NO level in BMEC upon various treatments.


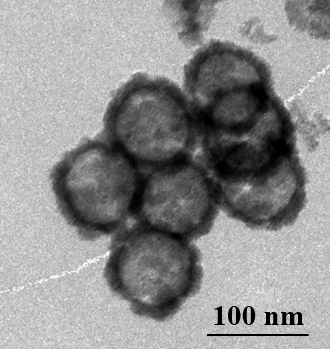


**Figure S3.** TEM image of CeO_2_@CO.


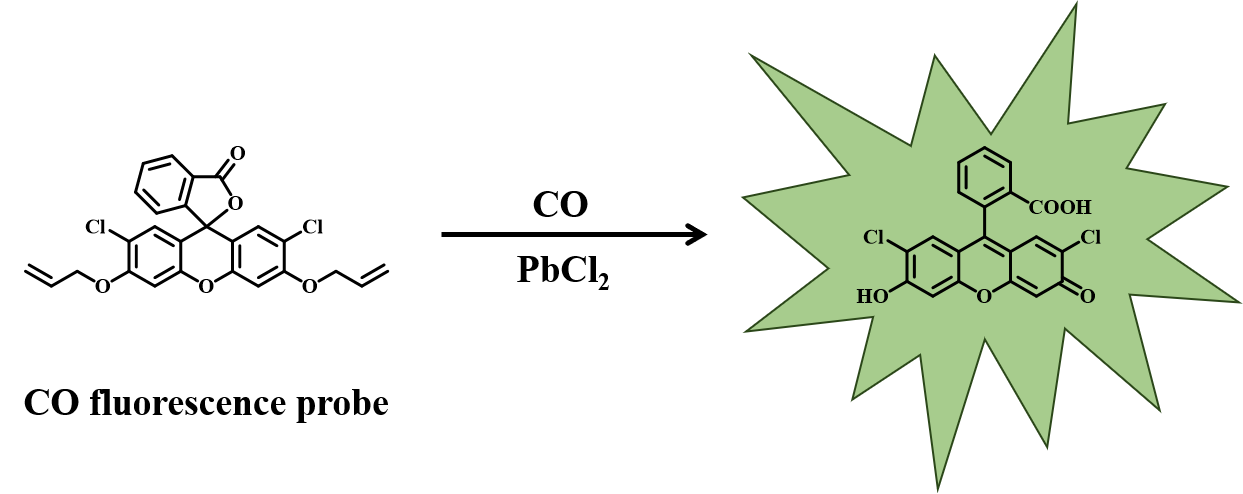


**Figure S4.** Illustration of CO fluorescence probe emitting green fluorescence in the presence of CO.


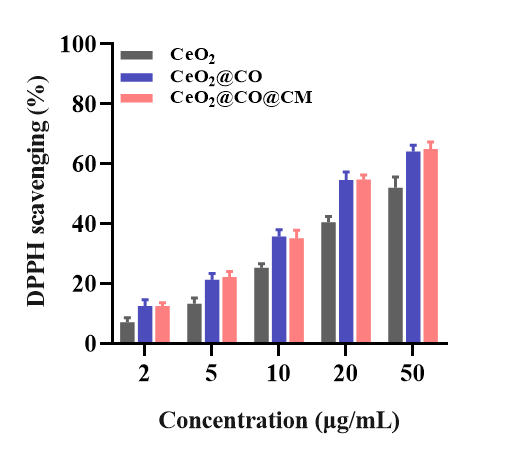


**Figure S5.** The 2,2-diphenyl-1-picrylhydrazyl (DPPH) assay to assess total antioxidant capacity of CeO_2_@CO@CM.


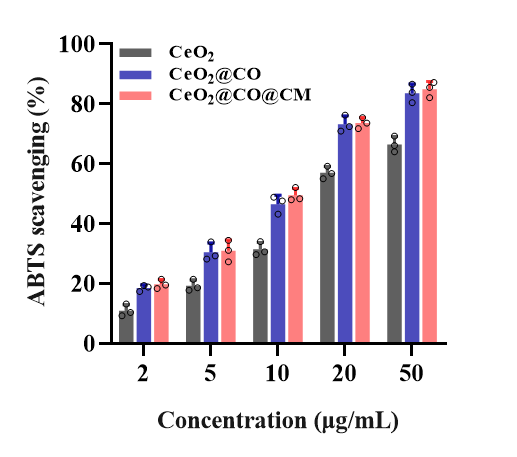


**Figure S6.** The 2,2' azobis (3-ethylbenzothiazoline-6-sulfonic acid) (ABTS) assay to assess total antioxidant capacity of CeO_2_@CO@CM.


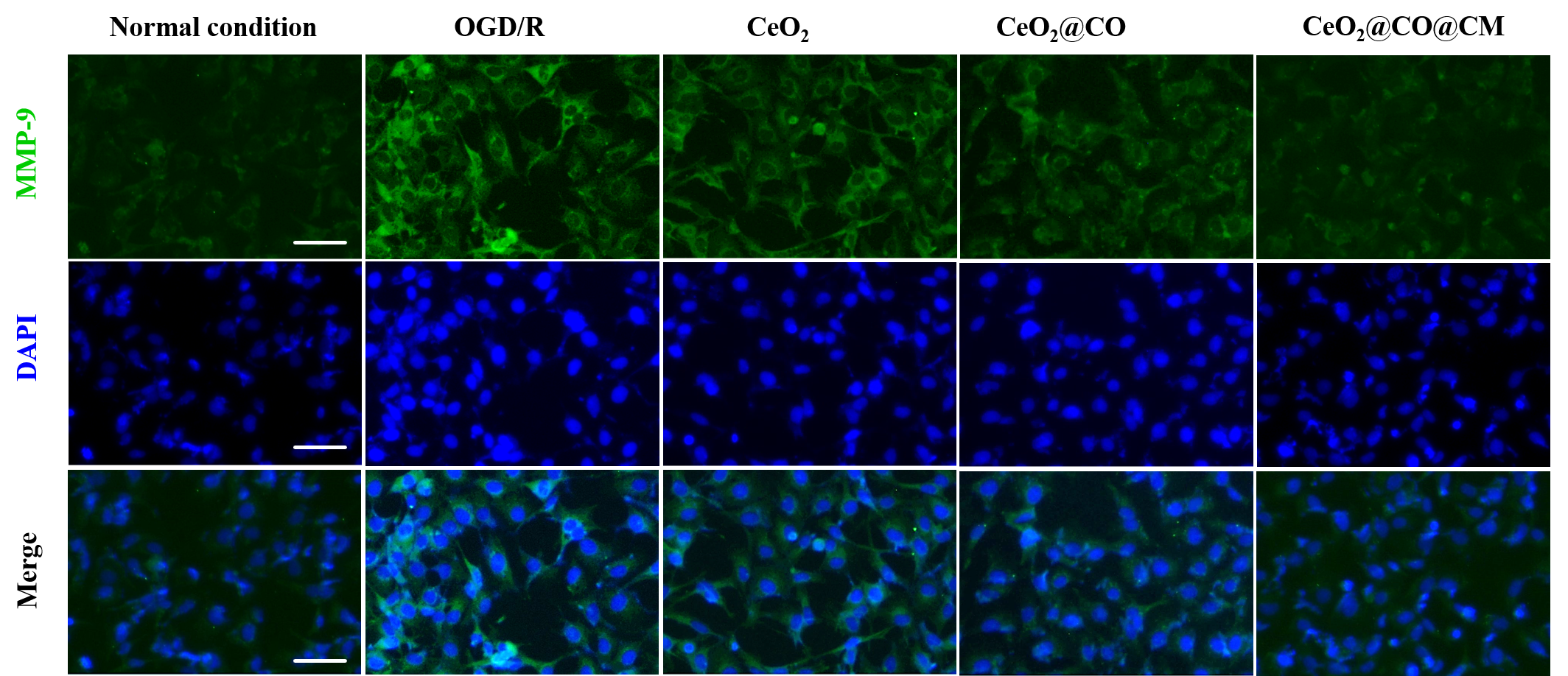


**Figure S7.** Immunofluorescence staining of MMP-9 in Bend.3 cells upon various treatments. Scale bars: 50 μm.


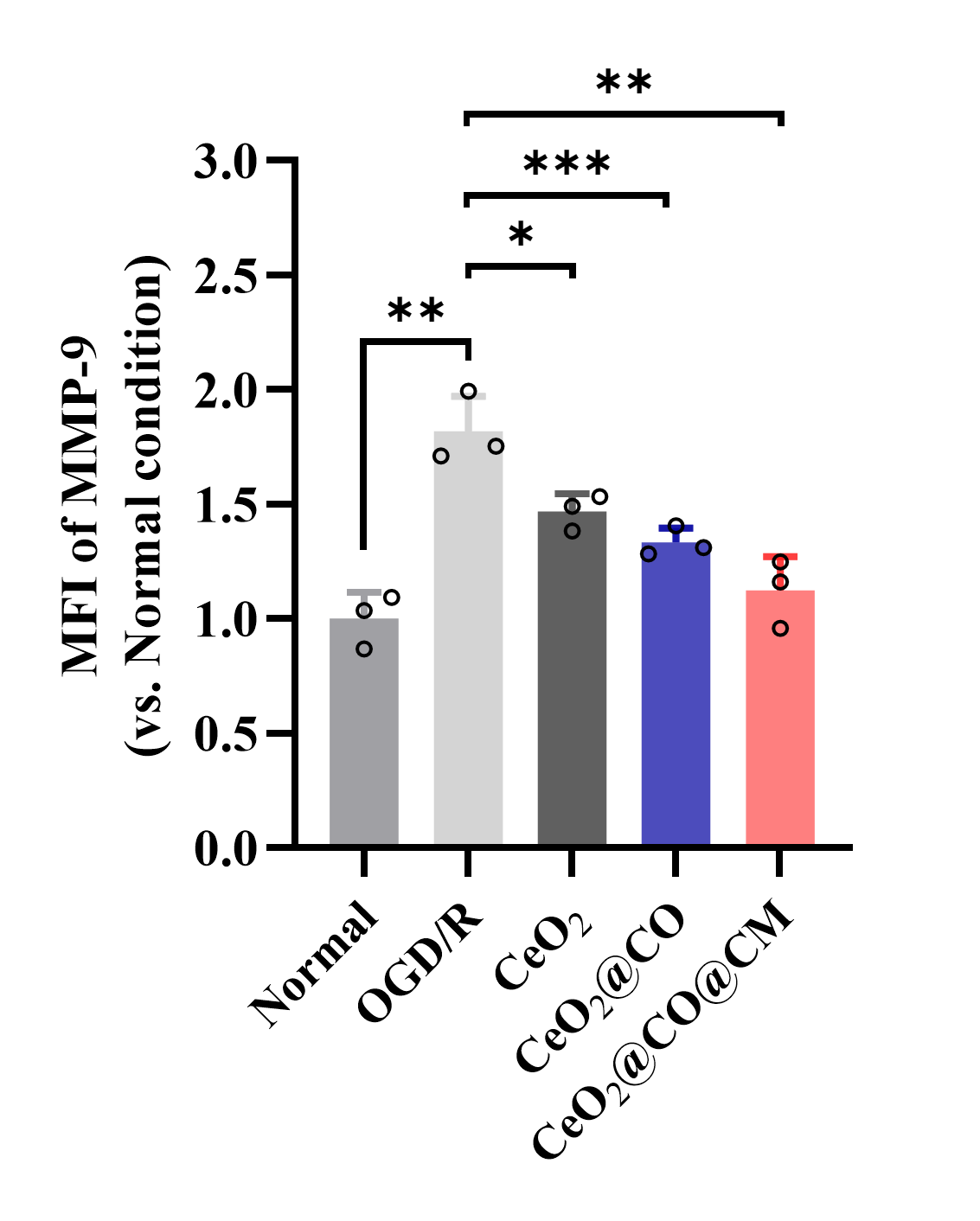


**Figure S8.** MFI of MMP-9 in Bend.3 cells upon various treatments.


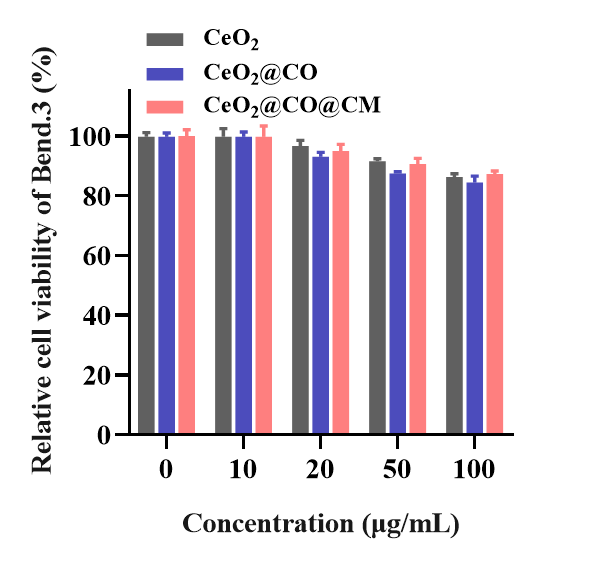


**Figure S9.** Cytotoxicity of CeO_2_@CO@CM to Bend.3.


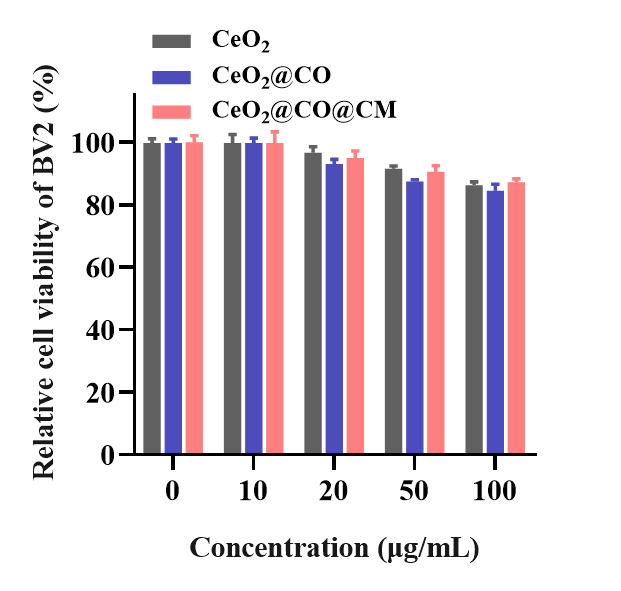


**Figure S10.** Cytotoxicity of CeO_2_@CO@CM to BV2.


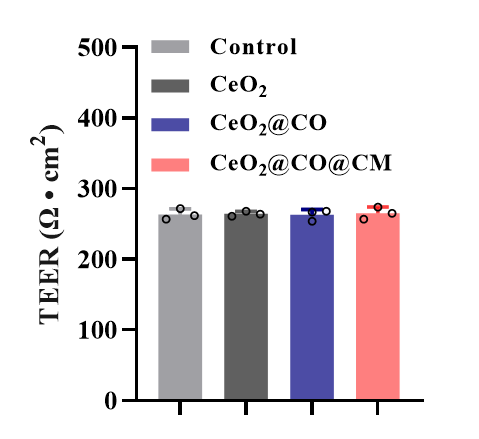


**Figure S11.** The trans-endothelial electrical resistance of the *in vitro* BBB model.


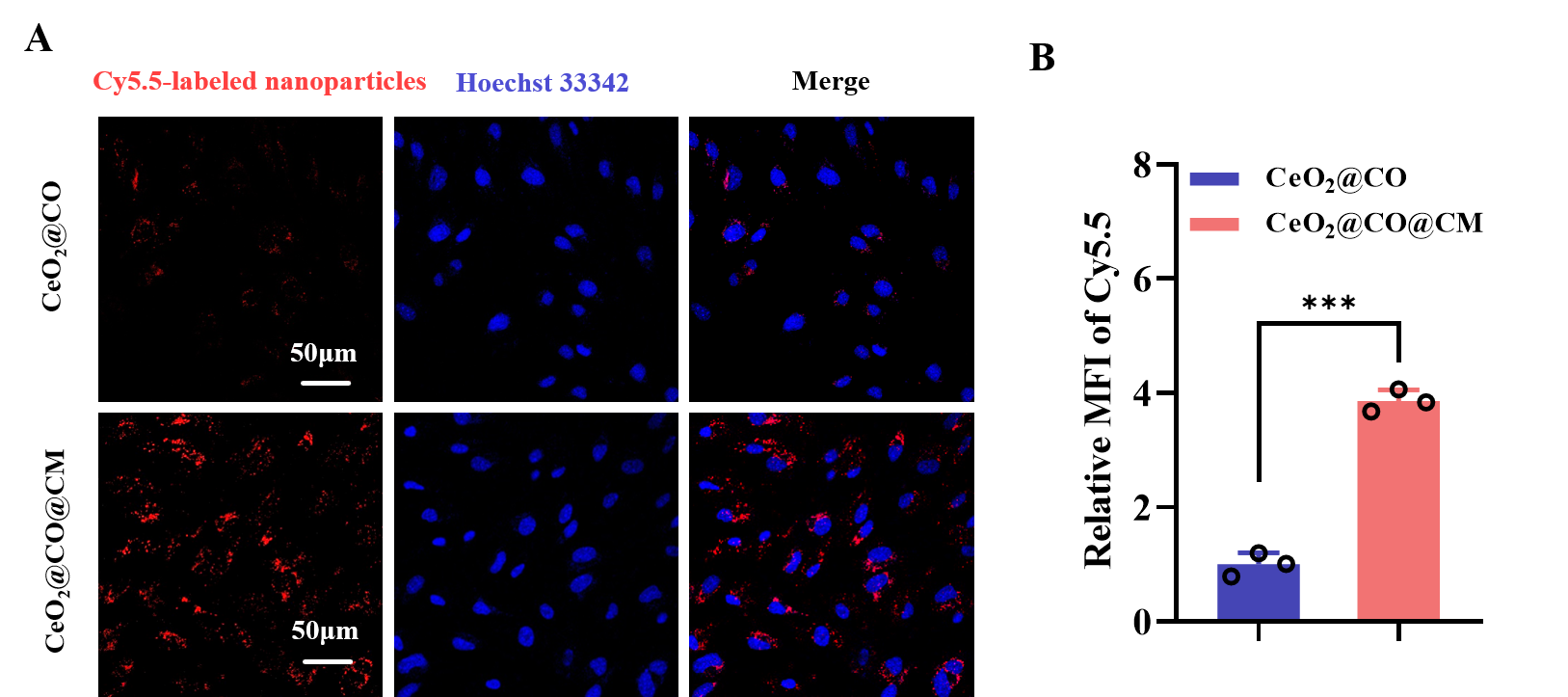


**Figure S12.** (A) CLSM visualizing cellular uptake of CeO_2_@CO and CeO_2_@CO@CM by HT22 cells after crossing the cell layer of Bend.3, (B) quantitative analysis of MFI reflecting the level of cellular uptake. Scale bars: 50 μm.


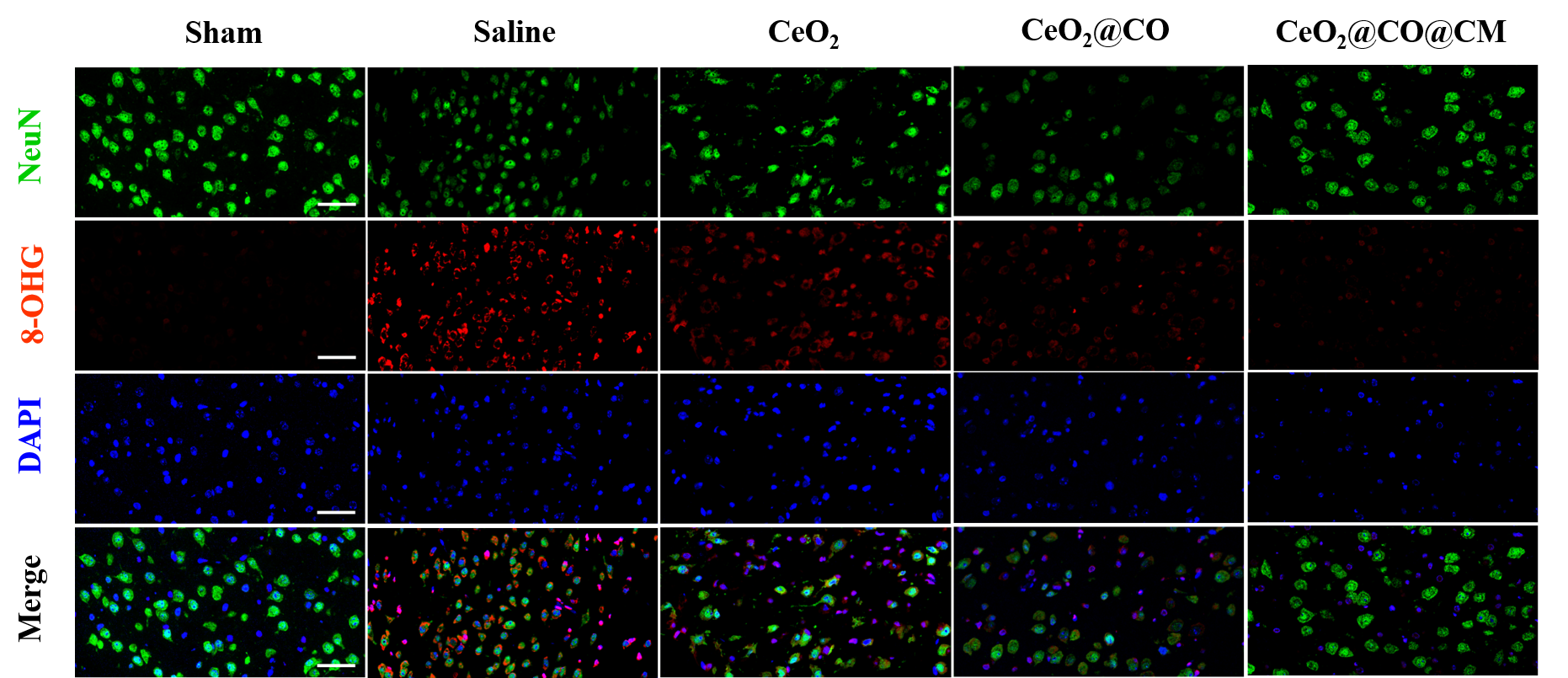


**Figure S13.** Representative immunofluorescence staining of 8-OHG (red)/NeuN (green)/DAPI (blue) showing oxidative damage in the penumbra upon various treatments at 24 h post-MCAO. Scale bars: 50 μm.


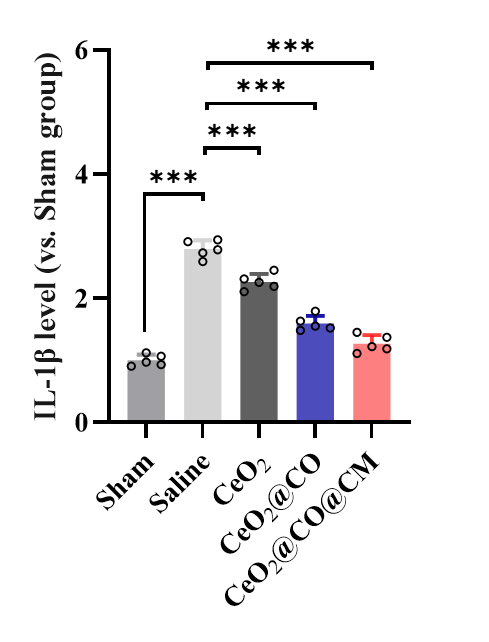


**Figure S14.** IL-1β levels upon various treatments in the penumbra at 24 h post-MCAO.


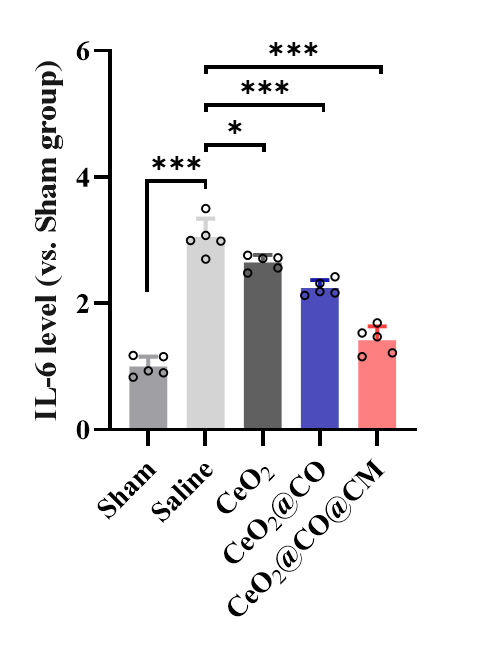


**Figure S15.** IL-6 levels upon various treatments in the penumbra at 24 h post-MCAO.


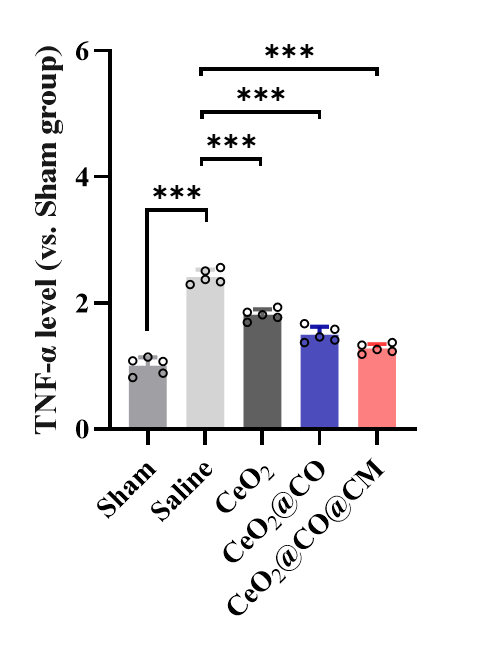


**Figure S16.** TNF-α levels upon various treatments in the penumbra at 24 h post-MCAO.


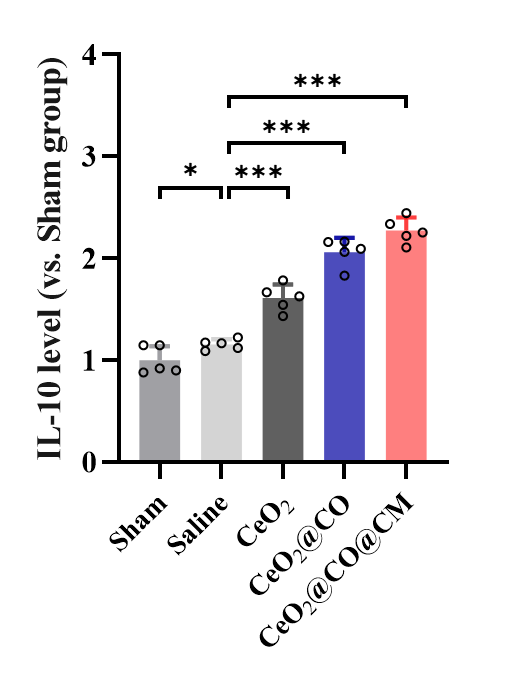


**Figure S17.** IL-10 levels upon various treatments in the penumbra at 24 h post-MCAO.


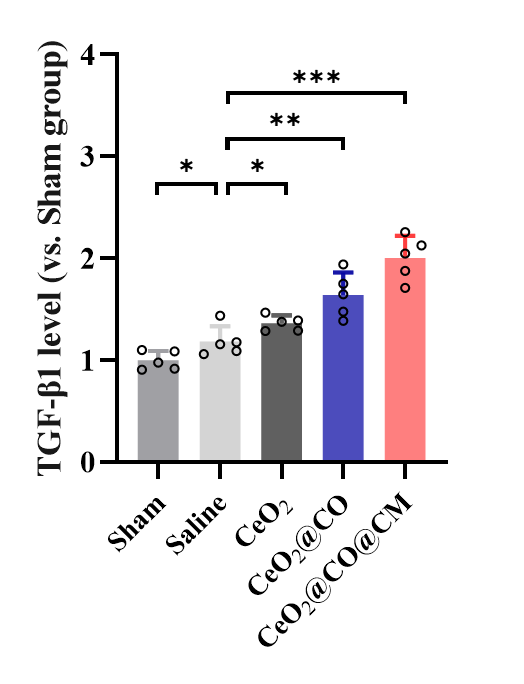


**Figure S18.** TGF-β1 levels upon various treatments in the penumbra at 24 h post-MCAO.


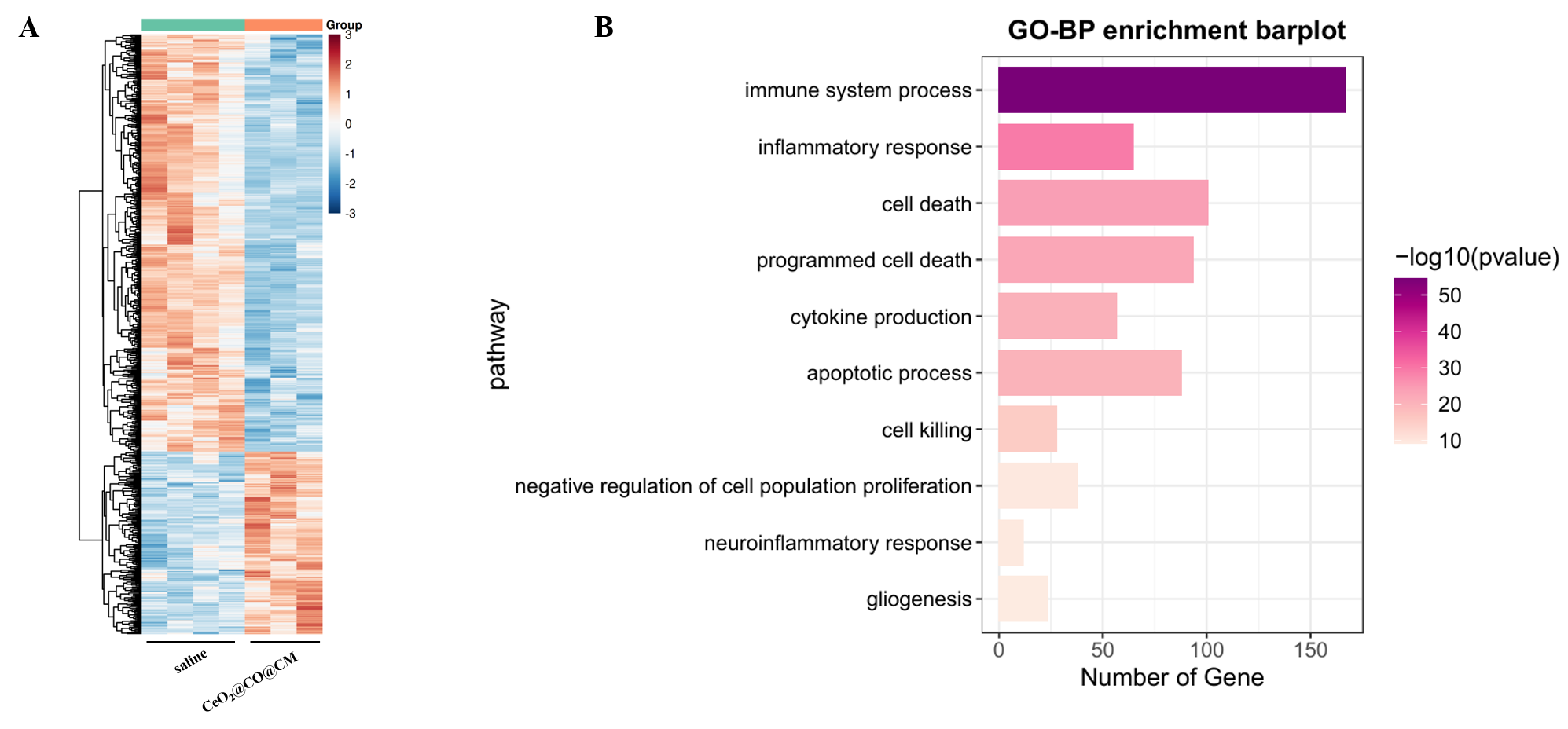


**Figure S19.** (A) Heatmap showing differentially expressed proteins between saline and CeO_2_@CO@CM treatment in proteomics results at 24 h post-MCAO. Protein expression values are scaled, with red indicating high expression and blue indicating low expression. (B) GO-BP enrichment barplot showing the significantly enriched pathways of upregulated proteins in saline-treated group compared to CeO_2_@CO@CM treatment group.


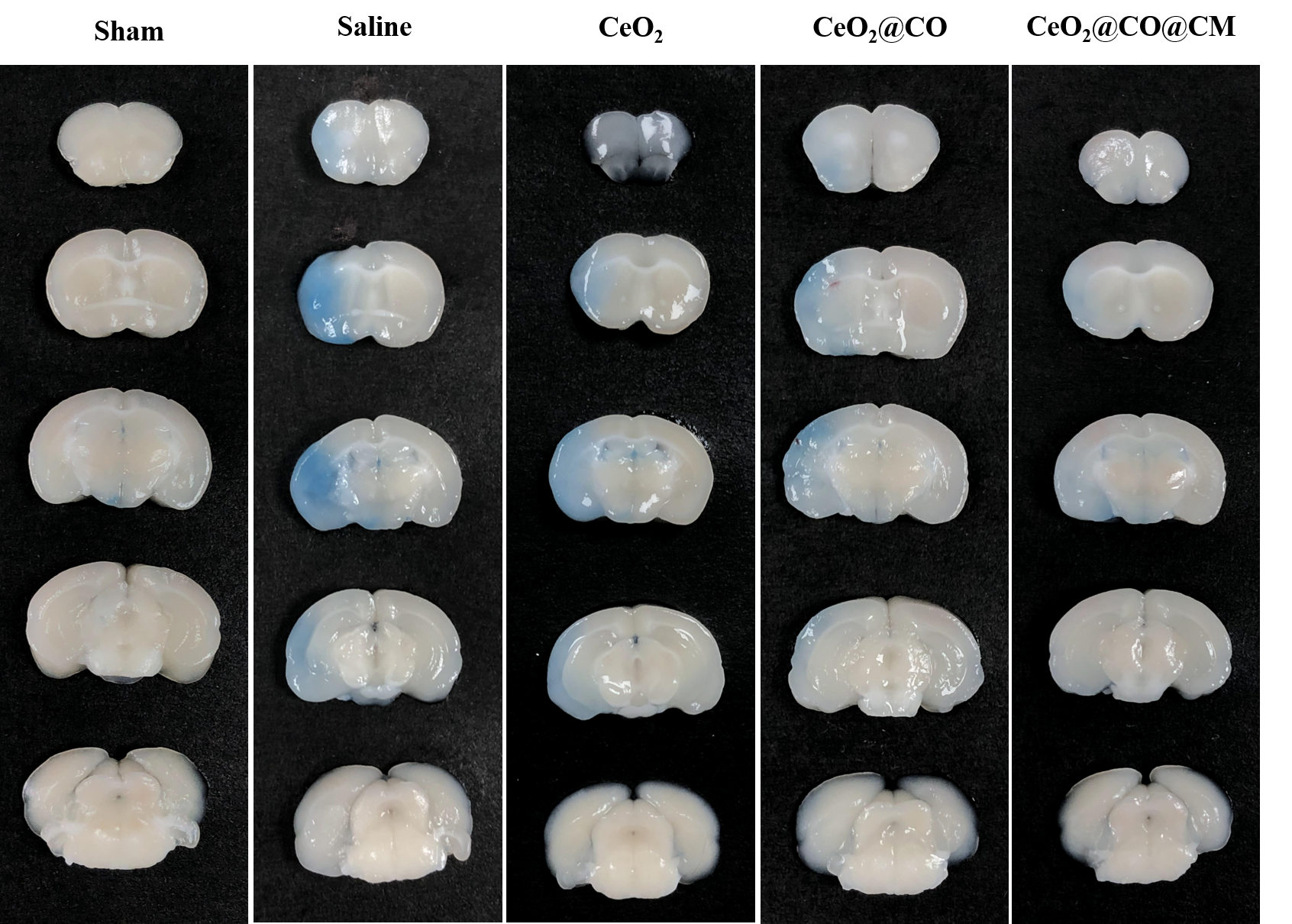


**Figure S20.** Representative Evans blue extravasation images of the brain upon various treatments at 24 h post-MCAO.


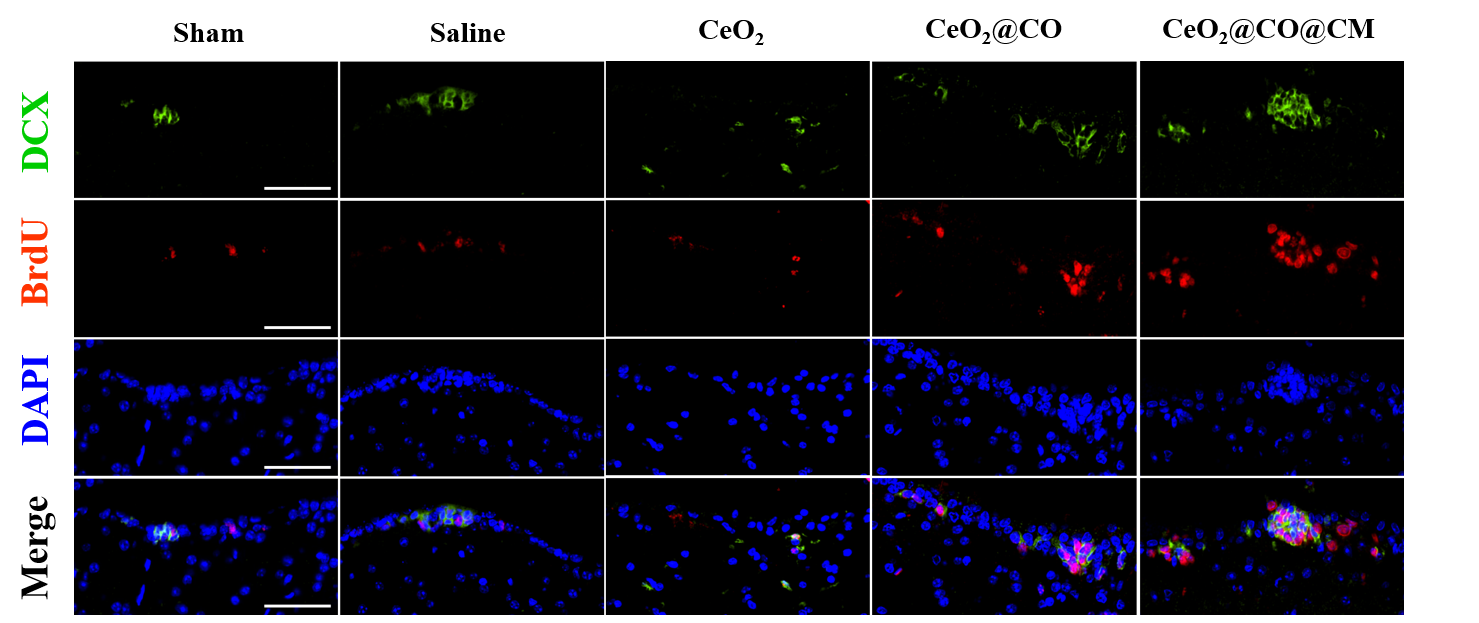


**Figure S21.** Representative immunofluorescence staining of BrdU (red)/DCX (green)/DAPI (blue) in SVZ upon various treatments at 14 days post-MCAO reflecting the proliferating neural precursor cells. Scale bars: 50 μm.


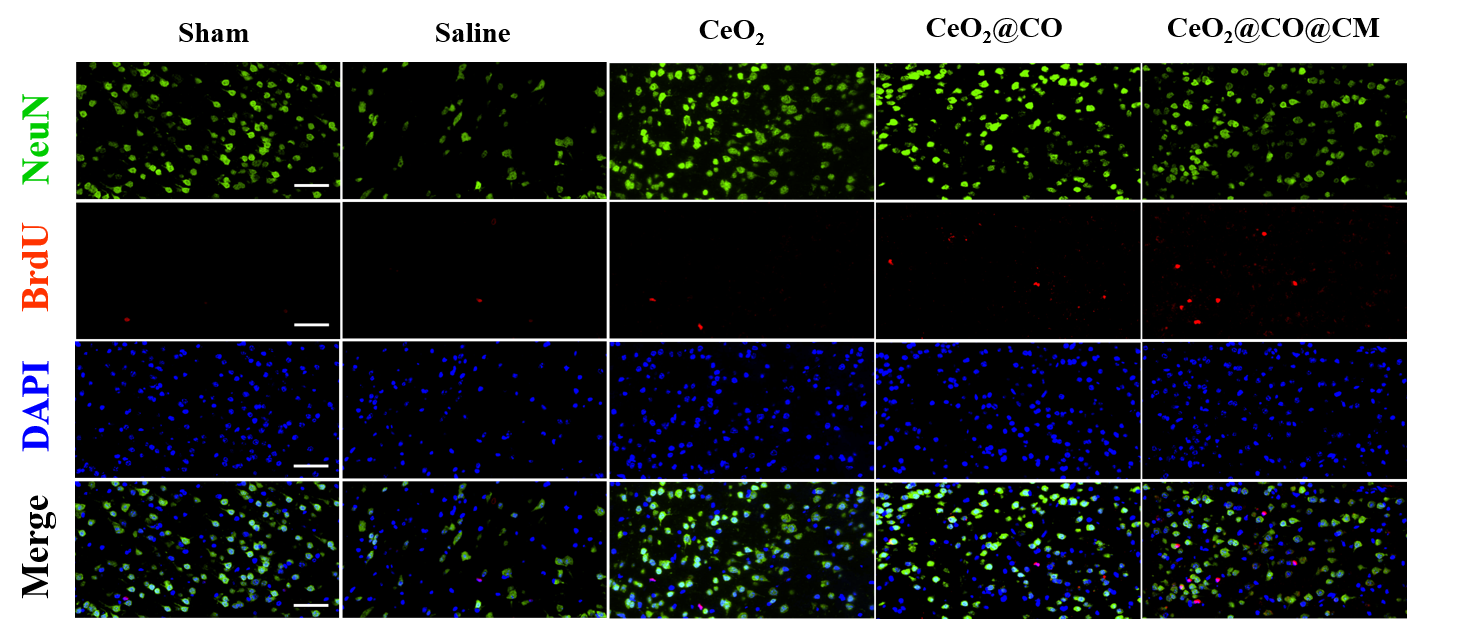


**Figure S22.** Representative immunofluorescence staining of BrdU (red)/NeuN (green)/DAPI (blue) upon various treatments in the penumbra at 14 days post-MCAO reflecting proliferating neurons. Scale bars: 50 μm.


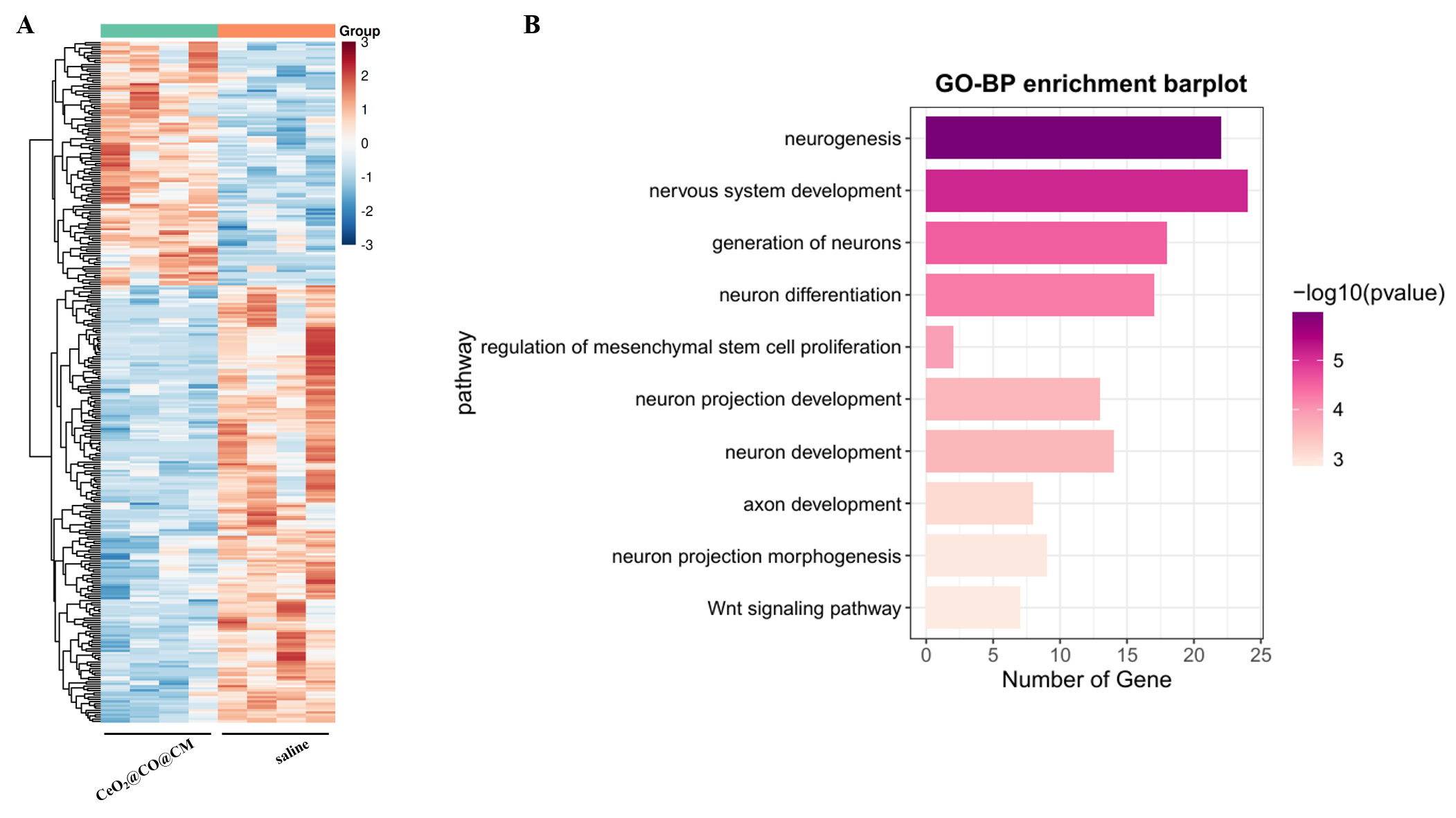


**Figure S23.** (A) Heatmap showing differentially expressed proteins between CeO_2_@CO@CM treatment and saline treatment in proteomics results at 14 days post-MCAO. Protein expression values are scaled, with red indicating high expression and blue indicating low expression. (B) GO-BP enrichment barplot showing significantly enriched pathways of upregulated proteins in CeO_2_@CO@CM treatment compared to saline treatment.


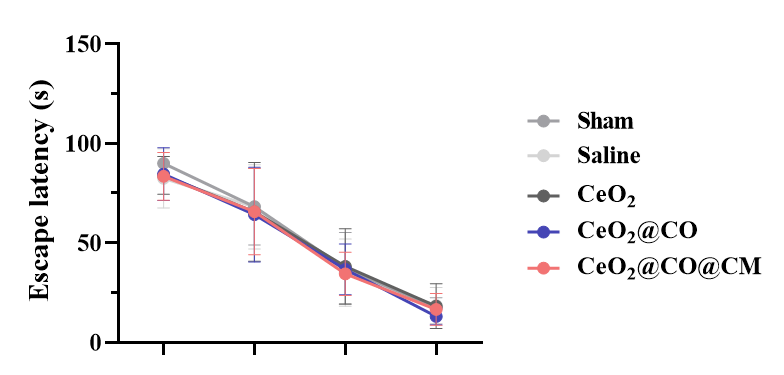


**Figure S24.** Escape latency of experimental mice in the training stage in MWM test.
